# Crystallizing the Uncrystallizable: Insights from Extensive Screening of PROTACs

**DOI:** 10.1021/jacs.5c07977

**Published:** 2025-07-23

**Authors:** Martin A. Screen, James F. McCabe, Sean Askin, Jamie L. Guest, Paul Hodgkinson, Aurora J. Cruz-Cabeza, Toby J. Blundell, Daniel N. Rainer, Simon J. Coles, Alexandra Longcake, Michael R. Probert, Clare S. Mahon, Mark R. Wilson, Jonathan W. Steed

**Affiliations:** † Department of Chemistry, 3057 Durham University, South Road, Durham DH1 3LE, U.K.; ‡ Early Pharmaceutical Development & Manufacture, Pharmaceutical Sciences, 468087 R&D, AstraZeneca, Macclesfield SK10 2NA, U.K.; § Advanced Drug Delivery, Pharmaceutical Sciences, R&D, AstraZeneca, Cambridge CB2 0AA, U.K.; ∥ School of Chemistry and Chemical Engineering, 7423 University of Southampton, Highfield Campus, Southampton SO17 1BJ, U.K.; ⊥ School of Natural and Environmental Sciences, Bedson Building, 5994 Newcastle University, Kings Road, Newcastle upon Tyne NE1 7RU, U.K.

## Abstract

PROTACs are new drug molecules in the beyond Rule of Five (bRo5) chemical space with extremely poor aqueous solubility and intrinsically poor crystallizability due to their structure, which comprises two distinct ligands covalently linked by a flexible linker. This makes PROTACs particularly challenging to understand from a solid-state preformulation perspective. While several X-ray structures have been reported of PROTACs in ternary complexes, to date no structures have been published of single component densely packed PROTACs, from which an understanding of PROTACs’ intermolecular interactions, and therefore physical properties, can be developed. An extensive crystallization protocol was applied to grow single crystals of a cereblon-recruiting PROTAC “AZ1” resulting in structures of an anhydrous form and a nonstoichiometric *p*-xylene solvate using 3D electron diffraction and synchrotron X-ray crystallography, respectively. The lattice energies are dominated by dispersive interactions between AZ1 molecules despite the presence of multiple hydrogen-bond donors and acceptors and planar aromatic groups, and both structures are built on similar intermolecular interactions. Thermal and spectral characterization revealed another solvate form containing dichloromethane. Amorphous solids produced by mechanochemical grinding of anhydrous AZ1 crystals also differed in dissolution characteristics from an amorphous solid produced by desolvating the dichloromethane solvate crystals, indicating that AZ1 may demonstrate *pseudo*-polyamorphism. This study paves the way for solid form screening and understanding in pharmaceutical systems that are far bRo5.

## Graphical abstract

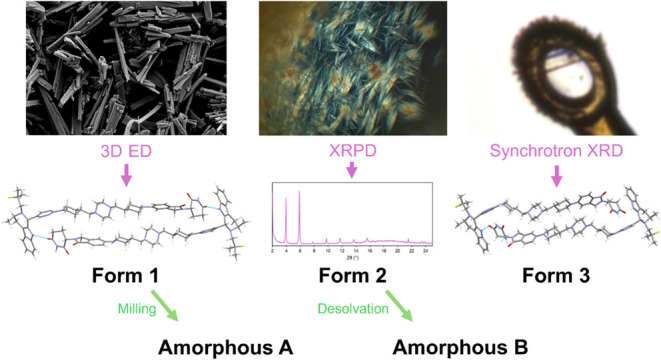

## Introduction

Formulation scientists in the pharmaceutical industry are responsible for ensuring that the active ingredients in drug products are bioavailable, and both physically and chemically stable. This requires the solid-state properties of the drug substance to be well understood and controlled. At least 50% of active pharmaceutical ingredients (APIs) subjected to industrial screening processes have been found to be polymorphic,^1^ meaning that they exist in more than one crystalline form depending on the intermolecular arrangements present. This presents both opportunities and challenges for the pharmaceutical industry: while different forms can be selected to optimize drug properties such as bioavailability, undesired transformations between forms can compromise product performance.^2,3^ Properties such as solubility and physical stability can vary greatly between different forms,^4^ which include polymorphs, solvates, salts and amorphous phases, so it is imperative to study the solid form landscapes of new drug compounds comprehensively before selecting the final form for drug product development. Identifying solid forms may also provide intellectual property opportunities.^5,6^ There are many experimental methods for discovering or isolating polymorphs as part of the screening process, including sublimation, crystallization from a single or binary solvent mixture, vapor diffusion, thermal treatment, slurrying, crystallization from the melt, changing pH, thermal desolvation of crystalline solvates, growth in the presence of additives, and grinding.^2^ Given the limits of time and resources, there is no guarantee that all possible polymorphs of a compound will be discovered or that the risk of unwanted transformations will be eliminated entirely after the screening process, but the majority of the thermodynamic and pharmaceutically relevant kinetic solid products are discovered by exposing the compound to a sufficiently wide range of crystallization conditions.^7^

The amorphous form of an API generally provides the greatest solubility and dissolution rate advantage since it is the highest energy solid state of a material often with greater molecular motion, and hence it has become of increasing interest to pharmaceutical industry.^3,8^ Amorphization is particularly interesting for poorly aqueous-soluble pharmaceutical compounds administered orally, where the inherently greater amorphous solubility and increased dissolution rate drive increased concentration within the gastrointestinal lumen.^8^ Amorphization of APIs has been implemented successfully within the pharmaceuticals industry and accounts for approximately 30% of drug products requiring solubility enhancement.^9^ However, amorphous API formulations are typically thermodynamically unstable and without physical stabilization they are prone to recrystallization, negating any dissolution benefits.^8^ Furthermore, amorphous solids are often more hygroscopic than their crystalline counterparts since their greater free volume allows water molecules to penetrate them more easily and the uptake of water plasticizes the solid, increasing molecular mobility and the likelihood of recrystallization.^10^ Numerous factors govern the physical stability of amorphous solids against recrystallization; those that depend on molecular structure are the aforementioned molecular mobility (correlated inversely with glass transition temperature *T*_g_), the configurational entropic barrier to crystallization, the enthalpic driving force to produce a solid form with lower configurational enthalpy, and the degree of hydrogen-bonding between molecules.^11,12^ Factors independent of the molecular structure include the humidity, mechanical stress, temperature and preparation method, since the thermal history of the material can vary the extent of molecular relaxation.^13^ Some organic compounds such as triphenyl phosphite can also exhibit polyamorphism, where distinct amorphous phases that vary in their local structures can be formed, often by using different methods to produce the amorphous phase.^14,15^ The first pharmaceutically relevant substance in which this behavior was noted was mannitol which can be prepared as two different amorphous phases at room temperature and pressure, one of which has substantially lower energy and density with a higher *T*_g_.^16^ Though rare, polyamorphism adds yet another layer of complexity to the solid-state landscape of a drug that can allow solid-state engineers to further tune the properties and behaviors of an API but equally requires one to characterize the amorphous phases produced in a variety of methods.

Targeted protein degradation (TPD) is an emerging therapeutic modality with the potential to tackle disease-causing proteins previously deemed “undruggable” with conventional small molecules, which are typically required to bind to a functional pocket on the protein to have a therapeutic effect. In contrast, TPD harnesses the cell’s own degradation machinery to eliminate target proteins, allowing it to modulate proteins regardless of whether they possess a suitable binding site, thereby significantly expanding the druggable proteome. In the 23 years since the conception of a proteolysis targeting chimera (PROTAC), a molecule capable of harnessing the ubiquitin-proteasome system to degrade a target protein, TPD has moved from academia to industry and is attracting substantial interest, with more than 10 PROTACs now in clinical trials.^17^ However, PROTACs are very poorly water-soluble and face challenges regarding their development into drug products with sufficient oral bioavailability. Aqueous solubility and cell permeability greatly impact the bioavailability of oral PROTACs in particular, such as those based on the E3 ligase cereblon (CRBN),^18^ and a poor understanding of the structure–property relationships for PROTACs makes it difficult to ensure that the degraders will reach their intracellular targets.^19^ The development of new drug compounds in general has expanded rapidly into a chemical space beyond Lipinski’s rule of 5 (bRo5),^20^ a guideline used to determine if a drug is likely to be orally active based on its chemical and physical properties,^21^ and the number of poorly water-soluble APIs has greatly increased in recent years with PROTACs included.^22^ Many physicochemical properties of PROTAC molecules, such as their molecular weight (MW), numbers of hydrogen-bond acceptors and donors (HBAs and HBDs respectively), and lipophilicity lie in the bRo5 chemical space since they require the functionality of two ligands and a linker group,^4,6^ and their poor aqueous solubility impedes various later stages of the drug development process.^18^ The high flexibility and size of PROTAC molecules increases the conformational space that they can explore, and most PROTACs are poorly crystalline as a result. While several crystal structures have been solved for ternary complexes between a PROTAC, a target protein and an E3 ligase,^23,24^ at the time of writing there are no published crystal structures of PROTACs alone to elucidate the intermolecular interactions between the drug molecules. Establishing preformulation data to aid in the development of solid PROTAC formulations such as their affinity for polymorphism, the relative stability of forms and their solvent content, is therefore a major challenge.

Compound “AZ1” is a PROTAC consisting of a lenalidomide-like ligand which recruits CRBN (referred to as the “CRBN-ligand”), and an estrogen receptor (ER) ligand (referred to as the “ER-warhead”) adjoined via a piperazine/piperidine-based linker moiety (Scheme 1). Compounds of this type selectively degrade ER α in several different breast cancer cell-lines including MCF-7, CAMA-1 and BT474.^25^ AZ1 is one of a PROTAC class that may provide greater ER degradation compared to current therapies and may be suitable for oral use as well as parenteral administration. The crystallizability, polymorphism and the stability of the amorphous form of AZ1 must be understood prior to developing a formulation approach. In this article we demonstrate the difficulty in applying conventional and high-throughput polymorph screening techniques to a cereblon PROTAC and present the first PROTAC crystal structures in the literature, alongside characterization of several other solid forms including potential *pseudo*-polyamorphs. This approach to understanding the solid forms landscape of AZ1 is the first PROTAC preformulation study of its type and illustrates the way in which solid form screening strategies need to be adapted using state of the art methodologies for this extreme bRo5 class of compound.

**Scheme 1:**
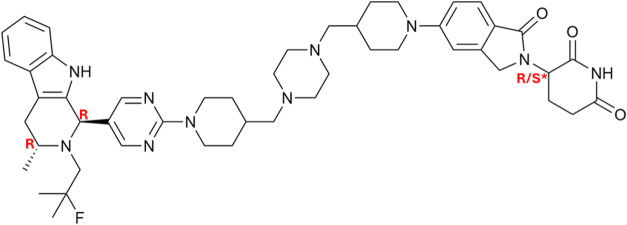
PROTAC Compound “AZ1”^a^

a Different diastereomer compositions of AZ1 are designated as AZ′8612 for a 1:1 mixture of RRR- and RRS-isomers (abbreviated AZ1_mix_), AZ′9929 for only the RRR- isomer (abbreviated AZ1_RRR_) and AZ′0163 for only the RRS- isomer (abbreviated AZ1_RRS_). The indole-containing moiety shown on the left constitutes the estrogen receptor (ER) ligand or “ER-warhead”. The imide-based moiety shown on the right constitutes the “CRBN-ligand”. The two are joined chemically via a piperazine/piperidine-based linker.

## Experimental Section

### Materials and General Methods

PROTAC compounds AZ′8612 (AZ1_mix_), AZ′9929 (AZ1_RRR_) and AZ′0163 (AZ1_RRS_) were supplied by AstraZeneca. All other chemicals and solvents were available from commercial sources and used without further purification. Infrared spectra were recorded between 4000 and 550 cm^–1^ using a PerkinElmer 100 FT-IR spectrometer with a μATR attachment. Powder X-ray diffraction (XRPD) patterns were collected at room temperature using a Bruker AXS D8 Advance GX003410 diffractometer with a Lynxeye Soller PSD detector, using Cu Kα radiation at a wavelength of 1.5406 Å and collecting from 2° ≤ 2θ ≤ 40°. Solution-phase ^1^H NMR spectra (400.20 MHz, DMSO-*d*_6_) were recorded at room temperature on a Bruker Neo-400 spectrometer, with chemical shifts reported in ppm relative to residual solvent signals (δ 2.50 for DMSO-*d*_6_).

### Crystal Screening of AZ1

Crystallization of AZ1 was first attempted by heating AZ1_mix_ in single solvents to produce supersaturated solutions upon cooling. The solubility of AZ1_mix_ at 1% w/v was assessed in 38 solvents spanning a broad range of polarity and boiling points. For solvents in which AZ1 was soluble at 1% w/v without requiring heat, the concentration was doubled by addition of powder until heat was required to dissolve (a full list of approximate solubility data is included in Supporting Information (SI) Table 1). Solutions at 1% w/v that required heat to dissolve but were stable for at least 4 h after heating were left undisturbed for 3 weeks. For solvents in which heat was required to dissolve AZ1 at 1% w/v but the resulting solutions were not stable for at least 4 h before precipitation, the concentration was halved by addition of solvent until solutions produced by heating were stable for at least 4 h. The concentrations of AZ1 required to produce such solutions was recorded. No dilutions were attempted in solvents which were insoluble even with heat at 1% w/v. All solutions were cooled passively to room temperature after heating. If no solid material was observed after 3 weeks, multiple repeats of the crystallization experiment were performed with different methods: cooling in a fridge or freezer, slow solvent evaporation, addition of antisolvent in 50 μL aliquots until translucent followed by reheating to dissolve and passive cooling, and antisolvent addition by vapor diffusion using diethyl ether. Solvents that produced crystalline material, identified by cross-polarized optical microscopy and XRPD, were studied further by controlled cooling experiments from 5 °C below boiling to −20 °C at 0.05 °C/min using a Cambridge Reactor Design Polar Bear Plus; slow evaporation of solvent from seeded solutions; and antisolvent layering in an NMR tube. Experiments that produced crystalline solids of AZ1_mix_ were also repeated using pure isomer samples of AZ1_RRR_ and AZ1_RRS_ to study their individual crystallizability.

### Encapsulated Nanodroplet Crystallization (ENaCt) Protocol

Stock solutions of AZ1_mix_, AZ1_RRS_ and AZ1_RRR_ in a range of 16 solvents varying in polarity and boiling point were prepared at near-saturated concentrations. In solutions with lower than 5.2 mg/mL solubility, the supernatant was used. Crystallization experiments were completed using an STP LabTech mosquito liquid handling robot using 96-well glass plates (SWISSCI LCP Modular, 100 μm spacer) and sealed with a 175 μm glass coverslip. An appropriate volume (typically 200 nL) of each oil was first dispensed onto the 96-well plates (aspirate 1.0 mm/min, dispense 1.0 mm/min), after which 50 nL of AZ1 solution was injected into each oil droplet (aspirate 20 mm/min, dispense 20 mm/min). Plates were then sealed with a glass coverslip, stored in the dark at room temperature for 14 days and inspected for crystal growth at regular intervals. Visualization of the experiment wells was carried out with a Nikon SMZ1000 microscope fitted with a cross polarizer. Photographs were taken with a GXCAM-U3–5 5.1MP camera. Full plate readouts are shown in Supporting Information Tables 2 and 3.

### Preparation of Form 1

A slurry of AZ1_mix_ (20 mg) in acetonitrile (5 mL) was stirred on an Expondo roller mixer at 100 rpm for 7 days, before the suspension was filtered and the isolated solid of Form 1 was obtained and characterized by XRPD, elemental analysis, FTIR, NMR, DSC, TGA and solid-state NMR. This form matches the powder pattern of AZ1_RRS_ as synthesized, which was also fully characterized.

### Preparation of Form 2

AZ1_mix_ (56 mg) was added to dichloromethane (0.5 mL) and heated to dissolve before cooling passively. After 3 weeks, needle crystals of Form 2 were afforded and characterized by XRPD, elemental analysis, FTIR, NMR, DSC, TGA and solid-state NMR. Isostructural needle crystals can also be produced by the same method but using AZ1_RRS_, with the same XRPD pattern, IR spectrum and DSC thermogram. Another isostructural sample of needle crystals can be prepared using the same cooling method from a chloroform solution.

### Preparation of Single Crystals of Form 3

AZ1_mix_ (5 mg) was added to *p*-xylene (0.5 mL) and heated to boiling before cooling passively. The sample did not dissolve and was left undisturbed for 11 months. One single crystal of Form 3 was afforded in a droplet of solvent on the vial wall.

### Electron Diffraction (3D ED)

3D electron diffraction data for Form 1 were collected on a Rigaku Synergy-ED (LaB_6_, 200 kV), equipped with a HyPix-ED hybrid pixel area array detector. The sample was gently ground between glass slides and a lacey carbon coated copper TEM grid (200 mesh; Agar Scientific, UK) dabbed in the solid. The grid was then plunged into liquid nitrogen and mounted on a Gatan Elsa cryogenic holder (model 698) and introduced into the column using cryo-transfer at 175(5) K. Data collections were conducted at 175(5) K in continuous rotation mode using a selected area aperture (∼2 μm diameter in the image plane) using CrysAlisPRO (version 1.171.44.78a). A range of particles was surveyed and a single data collection chosen for structure determination. The data were indexed, integrated and scaled using CrysAlisPRO (version 1.171.44.79a) without absorption correction to allow for dynamical refinement. The structure was solved using SHELXD^26^ and refined dynamically using olex2.refine (N-beam) implemented in the Olex2 (version 1.5-ac7-014)^27^ employing electron scattering factors.^28^ After initial refinement using the kinematical approximation, the obtained model was used as a starting model for dynamical refinement. All non-hydrogen atoms were refined using anisotropic atom displacement parameters, while hydrogen atoms were placed and refined based on geometry and using a riding model with distances fixed to neutron X-H bond lengths.^29^ Two reflections were omitted from the final refinement as they were considered untrustworthy based on their errors and disagreement with the general spread of reflections in the *F*_obs_ vs *F*_calc_ plot. Crystal data for Form 1: C_49_H_63_FN_10_O_3_, *M*_r_ = 859 g/mol, space group *I*2, *a* = 9.4516(8) Å, *b* = 6.2776(4) Å, *c* = 75.233(16) Å, α = 90°, β = 92.476(10)°, γ = 90°, *V* = 4459.7(10) Å^3^, *R*_1_ (*I* > 2σ­(*I*)) = 0.1539, *w*R**_2_ (all data) = 0.4140. Absolute structure was assessed using the Z-score method^30^ as implemented in Olex2, which gave Z-score values of 17.40 (noise-adjusted) and 9.19 (raw). Full crystallographic data, parameters of refinement and the hydrogen-bonding distances and angles are listed in SI Tables 4 and 5. Complete experimental and refinement information are contained in the deposited CIF along with structure factors and embedded. RES file. This structure is deposited in the CSD with CCDC reference code CCDC2445862.

### Single Crystal X-ray Diffraction (SC-XRD)

Single-crystal X-ray diffraction data for Form 3 was collected at 100.0(2) K on the I-19 beamline (Dectris Pilatus 2 M pixel-array photon-counting detector, undulator, graphite monochromator, λ = 1.0402 Å) at the Diamond Light Source, Oxfordshire and processed using Xia2/DIALS.^31–35^ The structure was solved by direct methods and refined by full-matrix least-squares on F^2^ for all data using Olex2 ^27^ and SHELXTL.^36^ All non-hydrogen atoms were refined with anisotropic displacement parameters. Hydrogen atoms were located on the difference map and refined isotropically on a riding model unless otherwise specified. The platon SQUEEZE routine was used to remove 0.75 solvent *p*-xylene molecules per asymmetric unit that could not be sensibly modeled due to disorder. Positional disorder was also observed for the lenalidomide end group atoms C1–5, N1, N2 O1 and O2 and refined to a ratio of 0.884(4):0.116(4) for the *R*- and *S*- enantiomers, respectively. Crystal data for Form 3: C_55_H_70.5_FN_10_O_3_, M_r_ = 938.71, space group *P*2_1_, *a* = 12.0183(5) Å, *b* = 6.2727(2) Å, *c* = 33.8422(11) Å, α = 90°, β = 99.389(3)°, γ = 90°, *V* = 2517.09(16) Å^3^, *R*_1_ (*I* > 2σ­(*I*)) = 0.0492, *w*R**_2_ (all data) = 0.1188. Full crystallographic data, parameters of refinement and the hydrogen-bonding distances and angles are listed in SI Tables 6 and 7. This structure is deposited in the CSD with CCDC reference code CCDC2448039.

### Preparation of Amorphous Solids

Amorphous form A can be prepared by adding AZ1_mix_ or AZ1_RRS_ to a 5 mL stainless steel grinding jar with one stainless steel grinding ball (6.4 mm diameter) and grinding with a Retsch MM200 Mixer Mill at 20 Hz for 15 min. The resulting solid has the same thermal and dissolution characteristics as AZ1_RRR_, which is amorphous as synthesized. Amorphous form B was prepared by heating Form 2 to 150 °C in an oven for 2 h to remove the solvent. Amorphous solids were characterized by FTIR, XRPD, DSC and TGA.

### Scanning Electron Microscopy (SEM)

SEM samples were prepared by adding solid powders to polycarbonate wafers and coating with 25 nm of platinum using a Cressington 328 Ultra High-Resolution EM Coating System. The images were obtained using a Carl Zeiss Sigma 300 VP FEG SEM microscope, operated at 5 kV using an in-lens detector.

### CrysIn Analysis

The AstraZeneca in-house developed Crystal Interaction (CrysIn) tool^37^ was used for quantification and comparison of static interactions between molecules in the Form 1 and Form 3 crystal structures. For each molecule in the asymmetric unit of the investigated crystal structure, all intermolecular pair (synthon) interaction energies within its first coordination shell were calculated using counterpoise corrected B3LYP-D3/6–31G­(d,p) molecular energies as implemented in Gaussian 16.^38^ This is a comparable approach as used in, for example, energy framework calculations in CrystalExplorer^39^ or PIXEL^40^ calculations.

### Cambridge Structural Database (CSD)

The single component drug predefined hitlist was retrieved from the CSD (version 5.45, Nov 23). The list contains 2388 refcode entries and contains redeterminations. Removing structural redeterminations within that list resulted into a subset of 1040 unique crystal structures. To facilitate computations, the set was further refined by removing structures with *Z*′ values other than one, and zwitterionic structures. This led to our final subset of 592 structures referred to as “CSD-drugs_NZ1,1_” throughout the manuscript. To summarize, the CSD-drugs_NZ1,1_ subset contains unique crystal structures (no redeterminations) of single component drugs crystallizing with *Z*′ = 1 and only nonzwitterionic compounds. This subset was used for further computational investigations.

### Lattice Energy Calculations for the CSD-drugs_NZ1,1_ Subset

Lattice (or cohesive) energy calculations were calculated for all structures in the CSD-drugs_NZ1,1_ subset using the Open Computational Chemistry (OCC) software.^41–43^ CIF files for the CSD-drugs_NZ1,1_ structures were retrieved from the CSD using a custom-made Python script which checks for hydrogen atom coordinates and adds them if missing with the method implemented in the CSD Python API. CIF files were used as input files for the OCC calculation. For each OCC calculation, the electron density of the drug compound is first calculated by retrieving its geometry from the CIF file and performing a single point energy calculation at the B3LYP/6–31 G** level of theory in the gas-phase.^44,45^ The electron density of the drug compound is then used to compute all drug–drug pairwise interactions within 30 Å of a reference molecule in the crystal structure. An overall lattice energy (*E*_latt_) is then computed by adding up all dimer interactions and dividing by two. While this is a simple way of estimating lattice energies and it certainly does not account for intramolecular energy penalties, it has been shown to reproduce benchmark DFT-d methods within 6.6 kJ/mol on average.^46^ Energies were computed successfully for 592 structures in the CSD-drugs_NZ1,1_ subset.

### Thermal Analysis

Differential scanning calorimetry (DSC) samples were prepared using Tzero standard pans and lids with pin-holes, and analyzed using a TA Instruments Q2000 differential scanning calorimeter by first equilibrating at 25 °C and heating to 400 °C at 10 °C/min. Amorphous samples were also analyzed at a heating rate of 50 °C/min. Samples analyzed in a heat–cool-heat cycle were first equilibrated at 25 °C, heated to 200 °C at 10 °C/min, cooled to 25 °C at 10 °C/min, and then heated to 400 °C at 10 °C/min. Modulated DSC samples were first equilibrated at 25 °C then heated to 200 °C, cooled to 25 °C and reheated to 400 °C using a modulated method with a scanning speed of 3 °C/min, an amplitude of ± 1 °C and a period of 60 s. The instrument was calibrated using indium standard prior to analysis, with a melting point onset of 156.89 °C and a heat capacity of 33.971 J/g. Thermogravimetric analysis (TGA) samples were analyzed using platinum pans and a TA Instruments Discovery thermogravimetric analyzer, heating from 25 to 400 °C at 10 °C/min.

### Solid-state Nuclear Magnetic Resonance (SSNMR)

Carbon-13 spectra were recorded at 125.72 MHz using a Bruker Avance III HD spectrometer and a 4.0 mm (rotor o.d.) magic-angle spinning probe. The spectra were obtained using cross-polarization, with a 4 ms contact time for CPTOSS experiments, at a sample spin-rate of 10 kHz. SPINAL-64 decoupling was performed on ^1^H with a 3 μs 90° pulse, for both CPTOSS and HETCOR experiments. Spectral referencing was with respect to tetramethylsilane (carried out by setting the high-frequency signal from adamantane to 38.5 ppm). The (indirect) ^1^H dimension for the HETCOR experiments was referenced by setting the high-frequency cross peak of glycine to 8.4 ppm, and scaling using the default for FSLG decoupling sequence.

### Hot Stage Polarized Optical Microscopy (HS-POM)

Thermomicroscopy analysis was performed using a polarized microscope (BX53, Olympus) coupled with the LTS420 hot stage (Linkam). Samples were heated from room temperature up to 190 °C and cooled back to 30 °C at a rate of 10 °C/min.

### Ultra Performance Liquid Chromatography (UPLC) Analysis

The concentrations of AZ1 were determined using a Waters ARC UPLC -MC206 system with an ACQUITY UPLC BEH C18 column (130 Å, 1.7 μm, 2.1 mm × 50 mm, Waters Corporation, UK) and a UV detection wavelength of 300 nm. The mobile phase of acetonitrile/water was varied in a gradient method from 95/5 v/v to 5/95 v/v at a flow rate of 1 mL/min.

### AZ1 Solubility

The thermodynamic solubility of AZ1 Form 1 in fasted state simulated intestinal fluid (FaSSIF) was determined by adding an excess of Form 1 powder to 1 mL of solvent and stirring at 1000 rpm for 24 h. The samples were then centrifuged for 30 min at 31,000*g* and the supernatant was diluted appropriately to maintain absorbance readings within the UPLC standard curve. The concentration of AZ1 was determined by UPLC analysis, converting peak area values to concentrations via a calibration curve. No difference in solubility between Form 1 prepared using AZ1_mix_ or AZ1_RRS_ was detected.

### Nonsink Powder Dissolution Measurements

Dissolution experiments were performed in triplicate for each sample. The powders were sieved using standard mesh sieves to remove particles larger than 150 μm. Vessels were charged with accurately weighed masses (approximately 1.3 mg) of the various crystalline and amorphous solids before adding the correct volume (approximately 10 mL) of prewarmed FaSSIF at 37 °C such that all slurries were accurately at ten times the measured solubility limit of Form 1. Slurries were stirred at 400 rpm for 2 h. Aliquots of the slurries were removed at each time point, centrifuged for 30 min at 31,000*g*, and the neat supernatant was analyzed to maintain absorbance readings within the UPLC standard curve. The concentrations of AZ1 were determined by UPLC analysis, converting peak area values to concentrations via a calibration curve. The pH of each dissolution slurry was recorded at the end of the experiment to confirm that it had not varied outside of the specification of the buffer.

## Results and Discussion

### AZ1 Crystallization

AZ1 contains three chiral carbon atoms, however in the present study, only the carbon atom denoted by an asterisk (*) in Scheme 1 may differ in configuration while the other two are fixed as R- configuration. AZ1 therefore comprises three distinct isomeric compositions: RRR-only (AZ′9929, abbreviated AZ1_RRR_), RRS-only (AZ′0163, abbreviated AZ1_RRS_), and a 1:1 mixture of both diastereomers (AZ′8612, abbreviated AZ1_mix_), all synthesized separately. X-ray powder diffraction (XRPD) analysis (Figure 1a) reveals that AZ1_RRS_ is crystalline with a powder pattern referred to henceforth as Form 1, while AZ1_RRR_ is amorphous. XRPD and solid-state ^13^C NMR (SI Figure S2) show that AZ1_mix_ matches the Form 1 structure of the AZ1_RRS_ sample but with broader powder pattern peaks and a slight hump in the baseline, yet a relatively crystalline NMR spectrum^47^ showing only slightly more disorder than the AZ1_RRS_ sample. This suggests that although both diastereomers are able to arrange in the Form 1 crystal structure, the presence of random stereoisomers and/or the small size of the crystal particles results in poor X-ray diffraction. Since AZ1_mix_ was by far the most abundant PROTAC sample available, most of the crystal screening was carried out using this sample and the separate RRS- and RRR- diastereomers were only screened using high-throughput methods that require low sample masses or in select experiments with a higher likelihood of success (Table 1).

**Figure 1:**
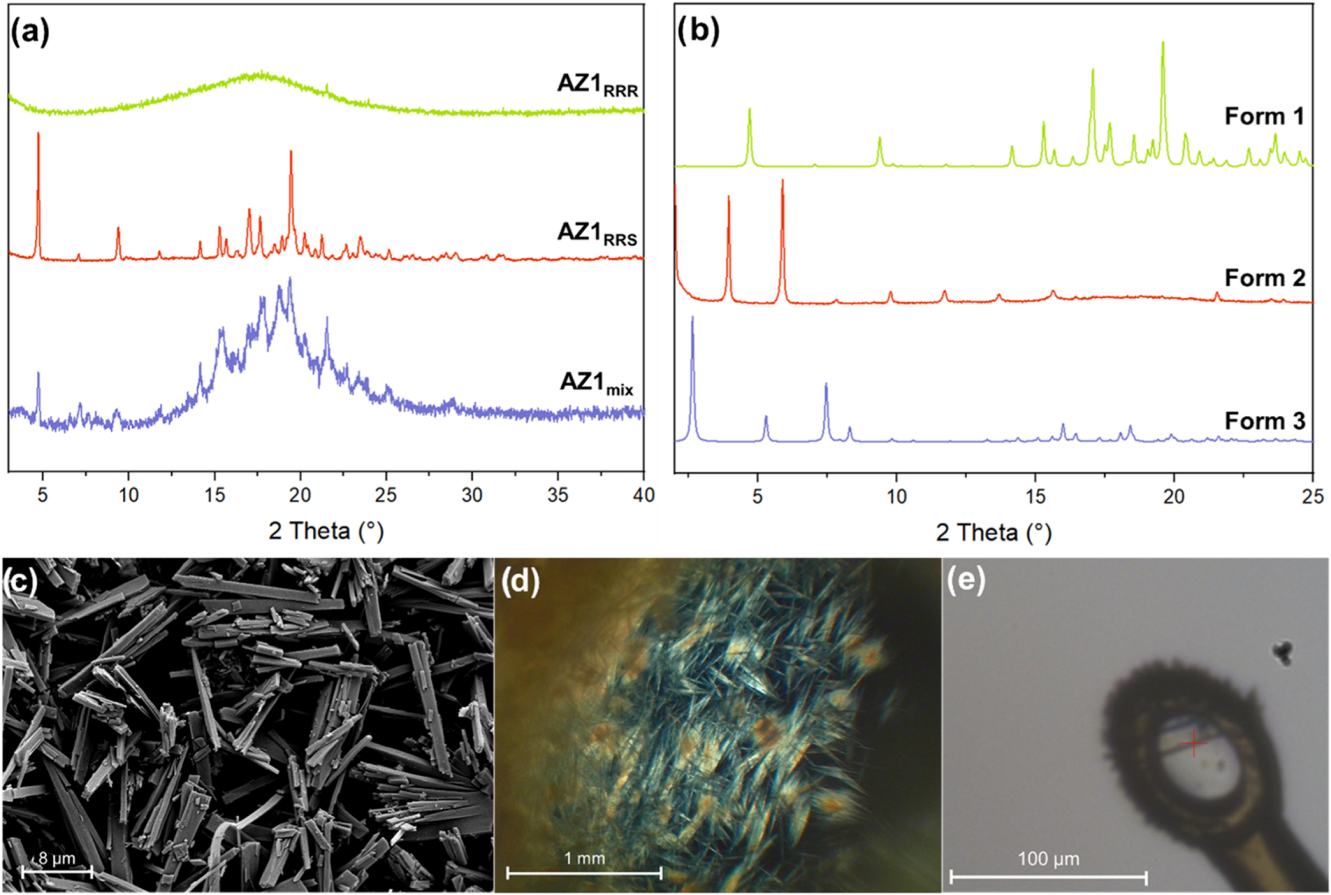
(a) XRPD patterns of AZ1_mix_, AZ1_RRS_ and AZ1_RRR_ as synthesized. (b) XRPD patterns of Form 1 (anhydrous), Form 2 (dichloromethane solvate) and Form 3 (p-xylene solvate). The powder pattern for Form 3 was simulated from SC-XRD data since there was not enough material to characterize by XRPD. (c) SEM image of Form 1 (AZ1_RRS_). (d) Optical microscope image of Form 2 (AZ1_mix_). (e) Diffractometer microscope image of Form 3.

**Table 1:** A Summary of the Structure and Compositions of AZ1 Solid Forms, and Their Method of Preparation^a^

**structure**	**composition**	**preparation**
form 1	AZ1_RRS_	as synthesized
	AZ1_mix_	slurry in MeCN
form 2	AZ1_RRS_	cooling crystallization in DCM
	AZ1_mix_	cooling crystallization in DCM
form 3	AZ1_mix_	cooling crystallization in p-xylene
amorphous type A	AZ1_RRS_	milling form 1
	AZ1_RRR_	as synthesized
	AZ1_mix_	milling form 1
amorphous type B	AZ1_mix_	desolvating form 2

a AZ1 solids may be composed of different isomers: RRR- only (AZ′9929, abbreviated AZ1_RRR_), RRS- only (AZ′0163, abbreviated AZ1_RRS_) or a 1:1 mixture of both (AZ′8612, abbreviated AZ1_mix_). Structures such as Form 1, Form 2 etc. are distinguished by their X-ray powder patterns, thermal and spectral analysis.

A thorough crystal screening process using a broad range of solvents and techniques was employed to attempt to grow single crystals of AZ1 and to study its potential polymorphism. At first, conventional crystallization techniques such as cooling of saturated solutions, slow solvent evaporation and antisolvent addition were attempted using AZ1_mix_. The high-throughput Encapsulated Nanodroplet Crystallization (ENaCt) protocol,^48^ which has been used successfully to grow single crystals of pharmaceutical compounds such as nifedipine,^49^ felodipine and cannabidiol,^50^ was also employed in the polymorph screening of AZ1_mix_, AZ1_RRS_ and AZ1_RRR_. Nanolitre droplets of AZ1 stock solutions in 16 solvents were dispensed inside of larger droplets of four inert, viscous oils and allowed to evaporate slowly alongside control droplets of stock solutions without oil.^48^ However, the only crystalline material isolated using the ENaCt method were two microcrystalline particles that did not diffract strongly enough for single crystal analysis using X-ray techniques, among hundreds of samples that were identified as amorphous residues based on a lack of birefringence and well-defined morphology. Eventually larger scale crystallization methods proved to be the only effective approach. Conventional crystallization experiments that produced crystalline samples of AZ1_mix_ were repeated using pure isomer samples of AZ1_RRR_ and AZ1_RRS_. Including the ENaCt experiments, a total of over 1,800 individual crystallization attempts were performed but fewer than 10 of these experiments produced a crystalline solid, with conventional passive cooling of supersaturated solutions and slurry in single solvents as the only successful techniques. Of these crystalline samples, only one was suitable for single crystal analysis using X-ray techniques.

Three distinct crystalline forms were identified from the crystalline solids produced: anhydrous AZ1 (Form 1 as previously described), which in addition to the as-synthesized AZ1_RRS_ material, can be produced as a crystalline powder by slurry of the initially semicrystalline AZ1_mix_ in acetonitrile; a dichloromethane solvate obtained as crystalline needles by cooling crystallization of AZ1_mix_ or AZ1_RRS_ from dichloromethane (Form 2); and just one crystal of a second solvate form obtained by cooling crystallization of AZ1_mix_ from p-xylene (Form 3) as shown in Figure 1e. The Form 3 sample took almost a year to grow but proved to be suitable for single crystal X-ray diffraction (SC-XRD). A sample that appears to be isostructural with Form 2 was also grown as needle-like crystals by cooling crystallization of AZ1_mix_ in chloroform. While Form 1 could be reproduced easily from either AZ1_mix_ or AZ1_RRS_, Forms 2 and 3 crystallized in only rare cases, proving the difficulty in exploring these compounds’ potential polymorphism and solvatomorphism.

SEM images reveal that the most crystalline Form 1 sample (AZ1_RRS_ as-synthesized) has a well-defined narrow plate morphology with particles ranging from approximately 10–15 μm in length and around 2 μm in width (Figure 1c). Optical microscope images of Form 2 crystals (Figure 1d) obtained from either AZ1_RRS_ or AZ1_mix_ reveal a needle-like morphology with a high aspect ratio and approximate length of 0.5–1 mm, however even the sample with the most crystalline powder pattern did not diffract X-rays well enough for structure determination even by synchrotron-source X-ray diffraction, and samples ground gently to a powder were unsuitable for analysis by electron diffraction, meaning that no structure solution was possible for Form 2. Since only one crystal of Form 3 was grown (Figure 1e), with a similar plate-like morphology to that of Form 1 but at a larger scale of 0.089 × 0.008 × 0.002 mm, any characterization beyond SC-XRD was not possible. Efforts to reproduce this form by numerous cooling and slurry crystallization experiments at a range of temperatures were unsuccessful. Slurries of Form 1 or Form 2 in p-xylene using magnetic stirring bars or impellers produced only amorphous powders within hours, whereas crystalline samples could be obtained by stirring slurries of Form 1 more gently using a roller mixer which produces much lower shear forces that grind the fragile crystalline particles into amorphous powder. However, XRPD analysis shows that after roller-mixing for several hours, days or months, only the form initially added or amorphous solid could be detected. Attempts to gradually cycle the temperature of a slurry containing Form 1 between room temperature and near boiling point, intending to reproduce similar conditions to the original experiment but with accelerated mass transport, produced no evidence of Form 3. Hence from the limited data available, we hypothesize that Form 3 is a metastable solvate in p-xylene. This demonstrates the poor crystallizability of PROTAC compounds and the difficulty not only in discovering new forms, but in reproducing them for characterization.

### Crystal Structure Analysis

Structure solution by 3D electron diffraction (3D ED) was possible for the as-synthesized AZ1_RRS_ crystalline powder. The crystal structure of Form 1 shows that AZ1 molecules form discrete hydrogen-bonded dimers aligned with the *a* axis via head-to-tail N9–H9···O1 interactions and aliphatic stacking (Figure 2). The dimers then stack along the *b* axis by numerous dispersive short contacts, and similarly along the *a* axis but to a lesser extent. The only interactions apparent along the *c* axis arise from close contacts between hydrophobic end-groups of neighboring AZ1 molecules. There is only one hydrogen-bond linking neighboring AZ1 molecules despite the availability of two hydrogen-bond donors and three carbonyl acceptors, leaving two that are involved only in weak interactions with C–H groups in adjacent molecules. Full interaction map analysis in Mercury^51^ (SI Figure S3) shows that all but one pair of potential hydrogen-bond donor and acceptor groups in the AZ1 molecule are not involved in interactions predicted by the map, suggesting that the crystal conformation and/or packing is unable to satisfy the hydrogen-bonding potential of all these moieties at once. XRPD analysis of the bulk sample is consistent with the calculated XRPD pattern from the single crystal data, confirming bulk solid form purity (SI Figure S4).

**Figure 2:**
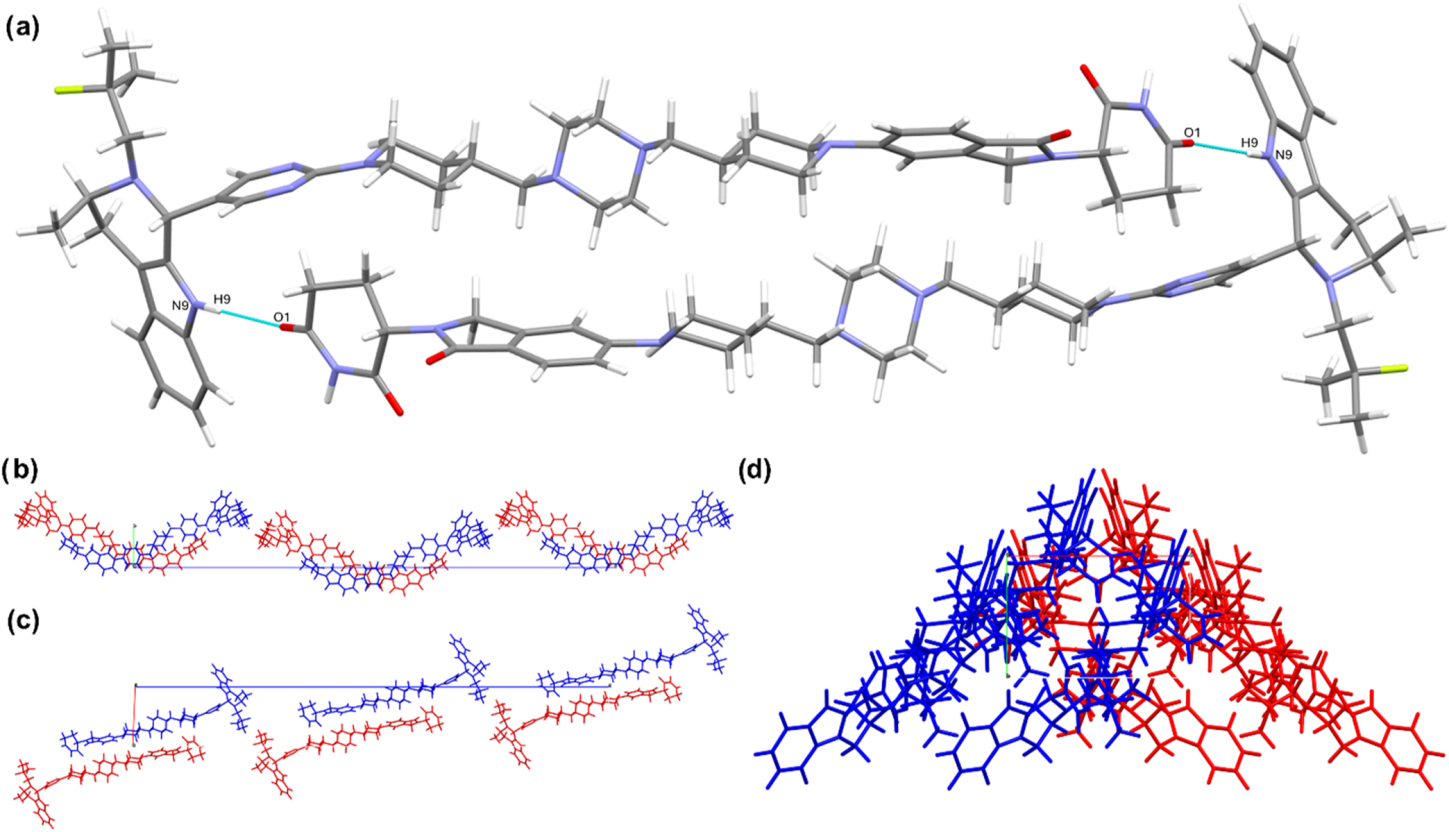
Form 1 crystal structure. (a) Dimer interaction between AZ1 molecules involving a head-to-tail N9–H9···O1 interaction. (b–d) Views down crystallographic *a*, *b* and *c* axes respectively.

Pairwise intermolecular interactions were evaluated using Crystal Interaction (CrysIn), a tool developed by AstraZeneca to quantify static interactions between molecules in the crystal using density functional theory (DFT).^37^ For each pairwise interaction between the asymmetric unit and its neighboring molecules, the interaction energy and its percentage contribution to the total lattice energy are calculated as well as a dispersive ratio, indicating the extent to which the interaction is dispersive or electrostatic in nature (a ratio of 1 describes a fully dispersive interaction, while 0.7 indicates some significant contribution of electrostatic attraction as well). The results of the calculation and the three pairwise interactions contributing the most to the Form 1 lattice energy are depicted in Figure 3, with the remaining pairwise interactions depicted in SI Figure S5. The results show that approximately 84% of the lattice energy can be attributed to four pairwise interactions with the greatest contribution from the formation of AZ1 dimers (d006, Figure 3b), through an interaction that is predominantly dispersive despite the presence of a hydrogen-bond. These dimers then pack along the *b* axis via dispersive aliphatic stacking interactions (d000/d001, Figure 3c). The next two strongest pairwise interactions consist of further dispersive stacking along the *a* and *b* axes, with only around 5% of the remaining lattice energy accounted for by interactions aligned with the *c* axis including an interaction between fluorinated moieties with slightly greater electrostatic contribution. This distribution of the lattice energy into interactions aligned with the three crystallographic axes is reflected in the plate-like BFDH morphology predicted by Mercury,^51^ where the longest crystal dimension is aligned with the crystallographic *b* axis, followed by the *a* then *c* axes, which is also in good agreement with the morphology observed by SEM. CrysIn analysis shows overall that besides the one hydrogen-bond present in the AZ1 dimer interaction, the crystal structure is dominated by the sum of many dispersive, aliphatic stacking interactions with little contribution from directional hydrogen-bonding or aromatic stacking interactions. The poor crystallizability of AZ1 may then be explained by the lack of strong and directional interactions that could guide the molecules into adopting the crystal conformation and pack into the three-dimensionally ordered structure, favoring instead an amorphous solid where the molecular conformation is less constrained and molecules may be able to make stronger local interactions at the expense of long-range order.

**Figure 3:**
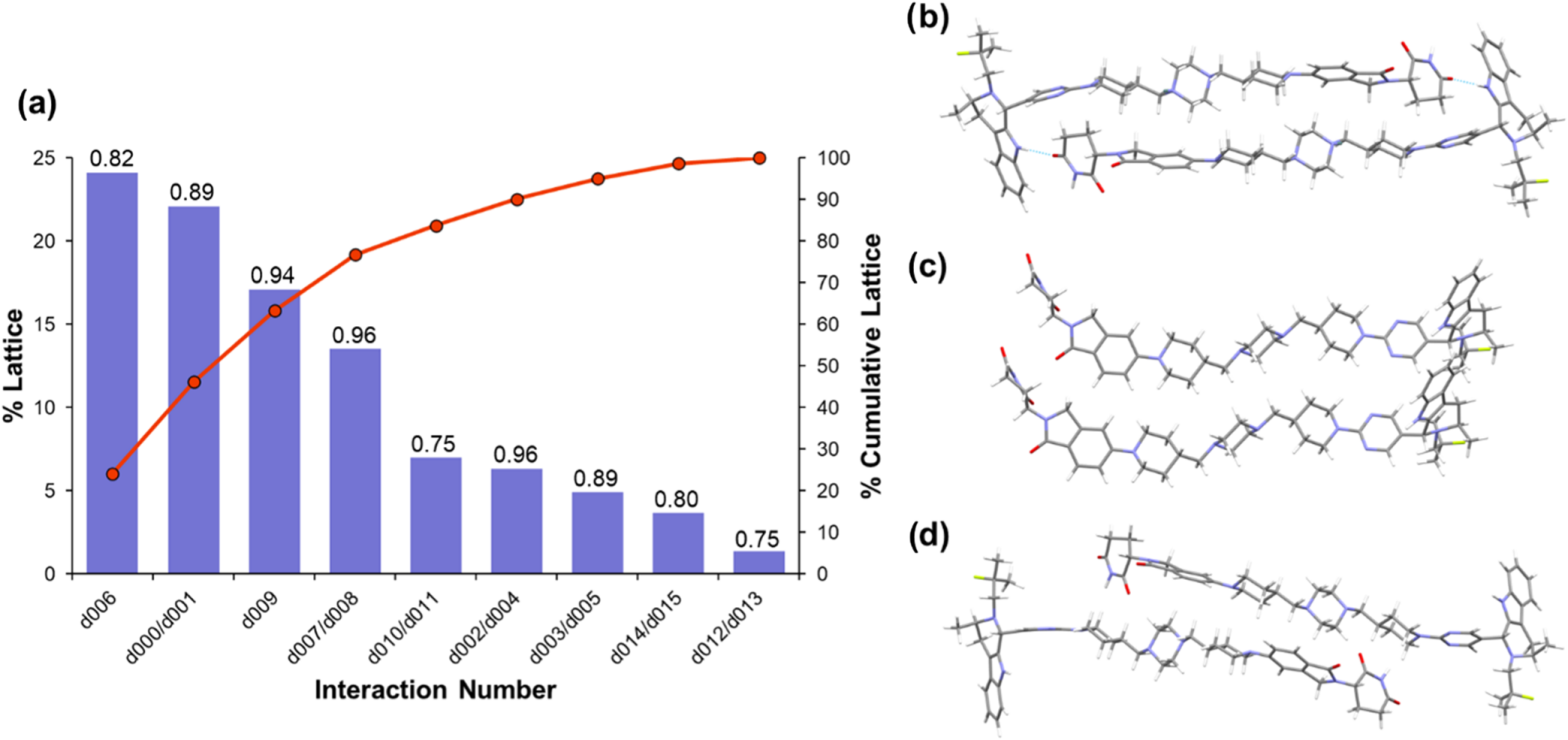
(a) CrysIn analysis of Form 1. The bar chart shows the percentage contribution of each pairwise interaction type to the total lattice energy. The line chart shows the cumulative lattice energy accounted for as pairwise interactions are summed together. The numbers above each bar chart are the dispersive ratios for each pairwise interaction, where a ratio of 1 indicates a fully dispersive interaction with no electrostatic contribution. (b–d) The three pairwise interactions that contribute the most to the Form 1 lattice energy by CrysIn analysis. From top to bottom: the AZ1 dimer interaction (d006); aliphatic stacking interactions along the *b* axis (d000/d001); aliphatic stacking interactions along the *a* axis (d009). The remaining six pairwise interactions are shown in SI Figure S5.

Only one crystal of Form 3 large enough for synchrotron-source SC-XRD was obtained via a cooling crystallization of AZ1_mix_ from p-xylene, in which AZ1 has very low solubility even at the solvent boiling point of 138 °C. Undissolved material at the bottom of the vial was identified as poorly crystalline Form 1 with a significant quantity of amorphous content, while Form 3 crystallized within a droplet of solvent high up on the side of the vial after approximately 11 months. We hypothesize that gradual mass transport up the sides of the vial and/or slow solvent evaporation over many months allowed a small quantity of AZ1 to recrystallize in this droplet on the vial wall. Structure solution of Form 3 reveals that it is a channel solvate with a 1:0.75 ratio of AZ1 to p-xylene, with the contribution of severely disordered p-xylene solvent which was removed from the structure solution using the SQUEEZE algorithm^52^ (Figure 4). The disorder in the CRBN-ligand of the AZ1 molecule (Figure 5a) arises from the presence of both RRR- and RRS- diastereomers in different unit cells since both isomers can pack into the same crystal structure, likely because the overall shape of the molecule is relatively similar for both configurations of this chiral atom, and because the intermolecular interactions are predominantly dispersive and isotropic in nature, unlike hydrogen-bonds, and so are not affected significantly by the difference in configuration. The RRR- and RRS- isomers were present in a ratio of 88% to 12% respectively in the crystal analyzed, despite beginning the crystallization experiment from a 1:1 ratio of the isomers. Hence, interestingly, the predominant diastereomer in Form 3 is different to the RRS isomer analyzed in the 3D ED structure of Form 1. It is unknown whether RRS-enriched crystals were also present in the sample since only one Form 3 crystal was suitable for analysis. However, the presence of other crystals consisting mostly of RRS- rather than RRR- seems plausible given that the most crystalline samples of Forms 1 and 2 produced in other crystallization experiments were grown using AZ1_RRS_ rather than AZ1_mix_. AZ1 molecules in Form 3 produce a hydrogen-bonded stack aligned with the *b* axis via head-to-tail N9–H9···O1 interactions. Along the *a* and *c* axes, AZ1 molecules interact only via dispersive short contacts. Like Form 1, the crystal predominantly consists of dispersive interactions with only one hydrogen-bond linking neighboring AZ1 molecules, despite the availability of two hydrogen-bond donors and three carbonyl acceptors. Full interaction map analysis (SI Figure S6) shows again that the molecular packing does not satisfy the hydrogen-bonding potential of all the available moieties.

**Figure 4:**
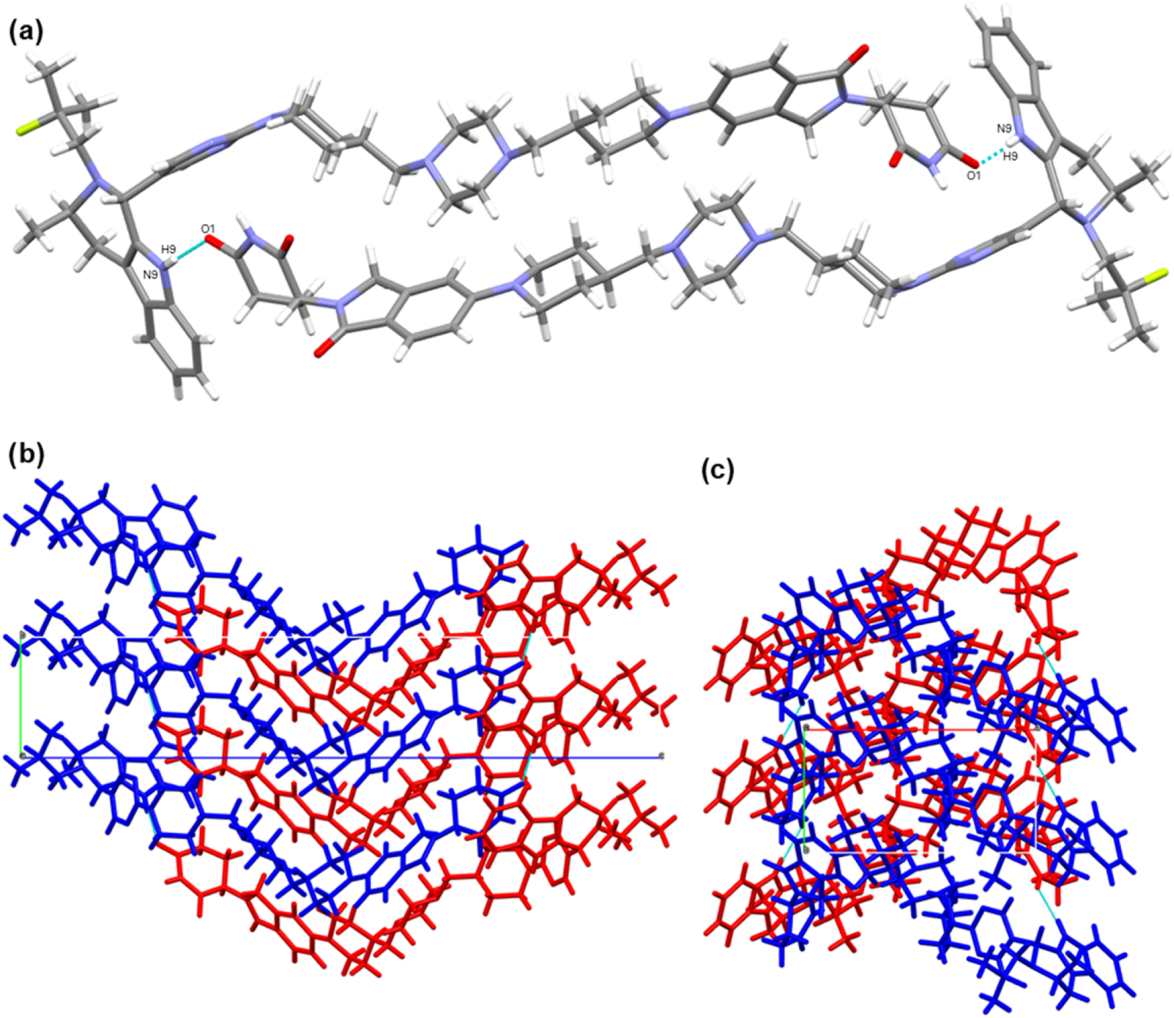
Crystal structure of AZ1 Form 3 with the disordered p-xylene solvent removed via the SQUEEZE algorithm. (a) The unit cell of Form 3 with a single hydrogen bond between the two AZ1 molecules, viewed down the *b* axis. (b) The view down the *a* axis. (c) The view down the *c* axis.

**Figure 5:**
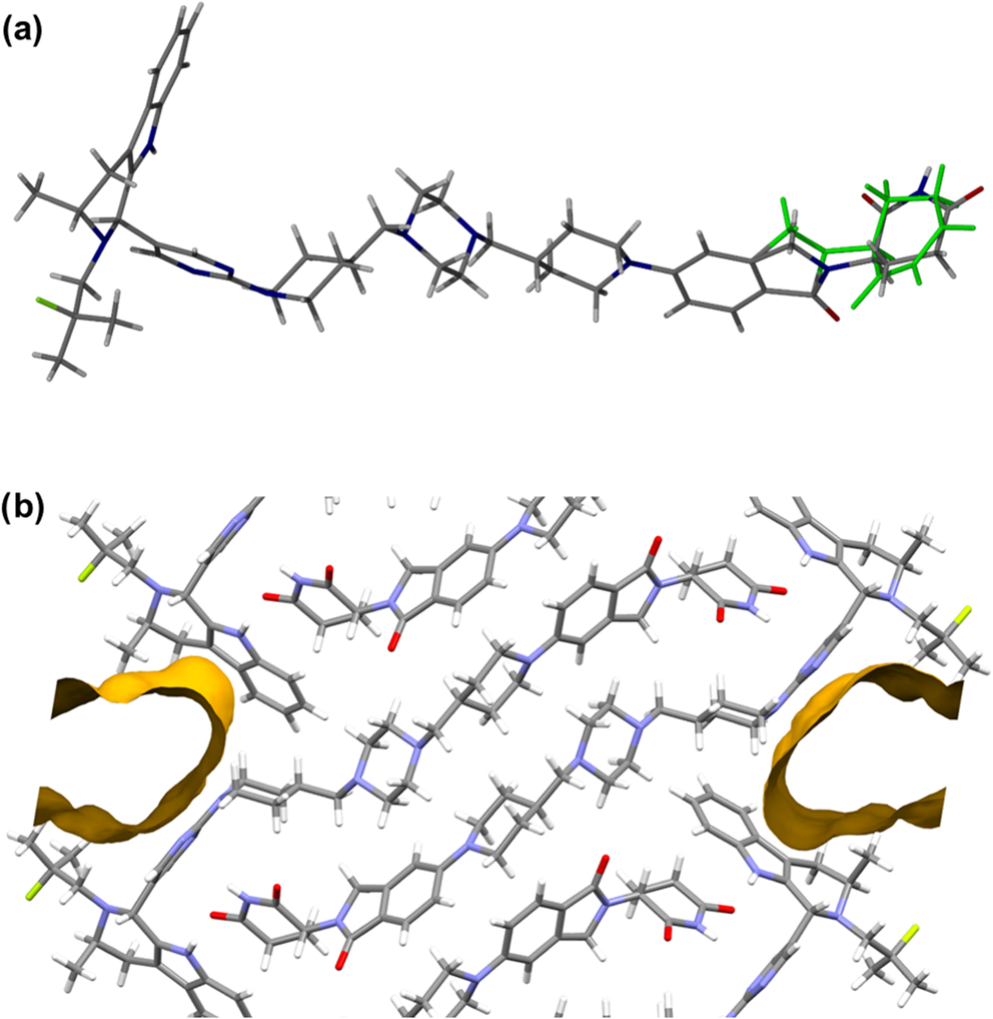
(a) Disorder in the CRBN ligand of AZ1 arising from the presence of both RRR- and RRS- diastereomers in an 88:12 ratio in the crystal structure of Form 3. The RRS- minor component is highlighted in green. (b) Solvent accessible voids in the crystal structure of AZ1 Form 3, depicted using Mercury.^51^

The solvent channels (Figure 5b) account for 14.7% of the unit cell volume (371.05/2517.09 Å^3^) and each unit cell contains 1.5 p-xylene molecules. The channel walls are hydrophobic in nature, consisting mostly of flexible alkyl groups and the faces of planar aromatic subunits of AZ1, such as the indole- and pyrimidine-like moieties in the ER-warhead. The Form 3 structure was also analyzed by CrysIn, noting the limitation that the p-xylene solvent is not explicitly included in the model and hence any contributions to the lattice energy from interactions between AZ1 and p-xylene are neglected. The 12% component of the RRS- isomer present as modeled disorder was also removed prior to the calculation, leaving only the RRR- major component. CrysIn analysis shows that approximately 85% of the lattice energy is accounted for by three pairwise interactions that are mostly dispersive in nature based on their dispersive ratio (Figure 6), with the remaining pairwise interactions depicted in SI Figure S7. The strongest interaction is dimer-like as in Form 1, showing a relatively high dispersive ratio again despite the presence of a hydrogen-bond. Unlike in Form 1 where the dimer interaction accounts for ∼24% of the lattice energy at −114.39 kJ/mol, in Form 3 the dimer accounts for ∼38% at −143.18 kJ/mol, with only a small difference between the dispersive ratios (0.82 in Form 1 compared to 0.78 in Form 3). The N9–H9···O1 interaction is roughly 0.1 Å shorter in the Form 3 structure but at a less linear N–H–O bond angle of 130° compared to 166° in Form 1. The stronger dimer interaction in Form 3 (AZ1_RRR_) compared to Form 1 (AZ1_RRS_) may result from an inherent difference in the ability of the two isomers to densely pack in the solid state. Like Form 1, stacking interactions aligned with the *b* axis dominate the Form 3 lattice energy. This demonstrates that despite containing mostly the RRR- diastereomer rather than the RRS- present in the Form 1 structure, the crystal structures are built from very similar intermolecular interactions.

**Figure 6:**
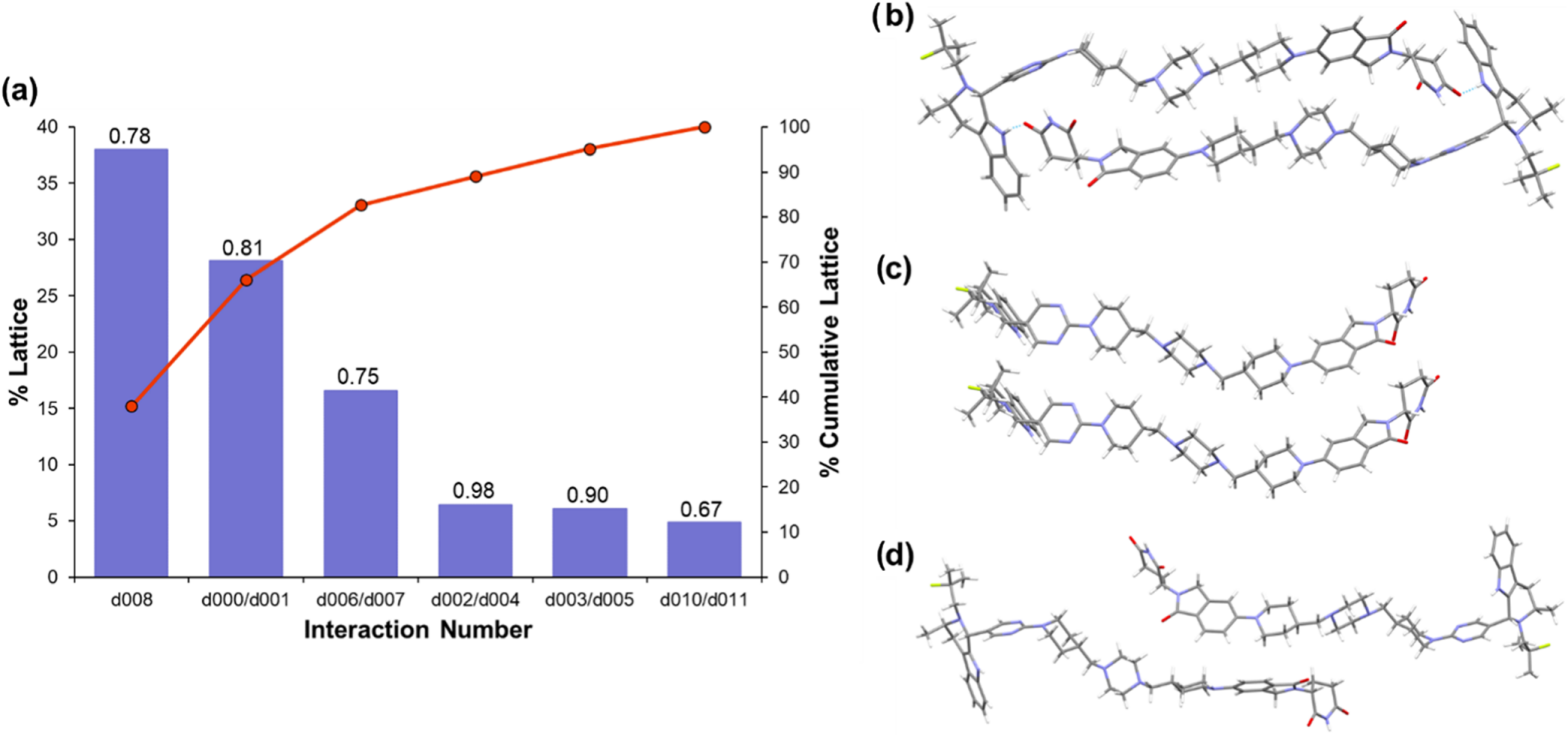
(a) CrysIn analysis of Form 3. The bar chart shows the percentage contribution of each pairwise interaction type to the total lattice energy. The line chart shows the cumulative lattice energy accounted for as pairwise interactions are summed together. The numbers above each bar chart are the dispersive ratios for each pairwise interaction, where a ratio of 1 indicates a fully dispersive interaction with no electrostatic contribution. (b–d) The three pairwise interactions of AZ1 molecules that contribute the most to the lattice energy of the Form 3 crystal structure. These three dispersive interactions cumulatively account for 84% of the lattice energy. From top to bottom: AZ1 dimer interaction (d008); aliphatic stacking interactions along the *b* axis (d000/d001); aliphatic stacking interactions in the *ac* plane (d006/d007). The remaining three pairwise interactions are shown in SI Figure S7.

The crystal conformation of AZ1 appears to be similar between Forms 1 and 3 regardless of the difference in diastereomer (Figure 7), adopting an elongated shape in both structures that allows a greater surface area of the molecules to stack closely in three dimensions, unlike a folded or C-shaped conformation that would impede the formation of many short dispersive contacts. As a large and relatively flexible bRo5 molecule with multiple rotatable bonds in its linker moiety, there is probably a considerable entropic penalty for AZ1 to adopt only the elongated crystal conformation present in either crystal. Coupled with a lack of strong, directional interactions such as hydrogen-bonds or aromatic stacking interactions in either crystal that could guide the molecule into adopting one conformation from the many available, the overall driving force for packing AZ1 into either of these crystal structures appears to be very weak. This explains the generally poor crystallinity observed in even the most crystalline samples obtained through the screening process, most of which were unsuitable for analysis by diffraction methods, and why many of the crystallization experiments using methods such as ENaCt, where solid material often precipitates within 2 weeks, were unsuccessful. The formation of only one diffraction-quality crystal of Form 3, 11 months after the initial cooling crystallization experiment was begun, further exemplifies these particularly slow crystal growth kinetics.

**Figure 7:**
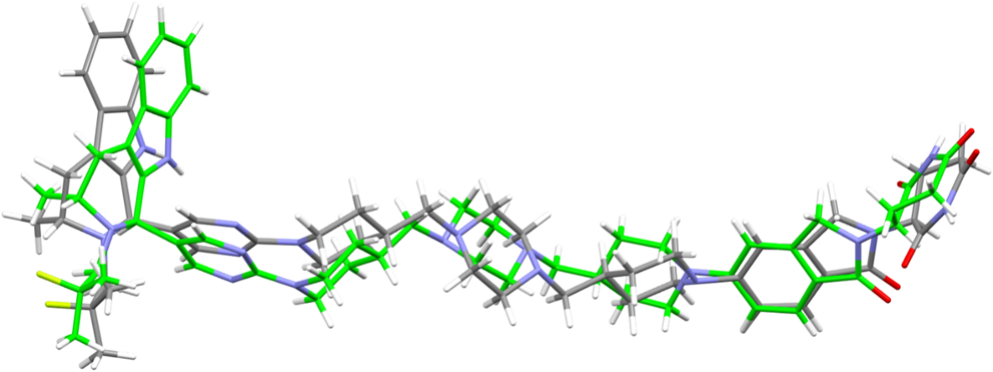
Comparison of AZ1 crystal conformations in Form 1 (full color) and Form 3 (green highlight). Only the major RRR- isomer component of the Form 3 crystal structure is shown. Only the RRS- isomer is present in Form 1.

### Comparison to Crystalline Drugs in the CSD

The Cambridge Structural Database contains published solid forms of more than 785 unique drug molecules,^53^ providing the opportunity to compare the available PROTAC crystal structures of AZ1 Forms 1 and 3 with a broad range of API compounds for which crystal data already exists. The CSD search protocol is described in the Experimental section and produced a subset of 738 single component crystal structures with *Z*′ = 1 from the 2388 available entries, designated “CSD-drugs_NZ1,1_”. The experimental also describes a computational method to calculate lattice energies and pairwise interaction energies for all structures including Forms 1 and 3. As with the CrysIn analysis, it should be noted that the removal of disordered p-xylene solvent and the minority AZ1 diastereomeric component will affect the computed energies in the Form 3 structure.

The computed lattice energies of the crystals containing drug compounds in the CSD (CSD-drugs_NZ1,1_ subset) are shown in Figure 8. Typically, the lattice energies of the nonzwitterionic drugs become more stabilizing the larger the compound. Remarkably, Form 1 has lower lattice energy (−373.6 kJ/mol) than all CSD-drugs_NZ1,1_ compounds with Form 3 being only about 8 kJ/mol less stable (−365.3 kJ/mol), not including any further stabilization from interactions between AZ1 and the unmodeled p-xylene solvent. This suggests that the particularly low aqueous solubility of AZ1, and potentially other PROTACs, is due to not only their lipophilicity but also their very strong lattice energies, giving them some degree of both “brick-dust” and “grease-ball” solubility characteristics. We also note that only one structure in the CSD-drugs_NZ1,1_ subset contains a drug molecule larger than AZ1; this corresponds to the drug compound Sirolimus, also known as Rapamycin.^54^ Of the crystal structures with a lattice energy lower than −300 kJ/mol and/or containing a compound with >100 atoms, all but one are structures of bRo5 molecules (with ganciclovir as the exception, CSD refcode UGIVAI01). This group of structures consists of the drug compounds digoxin, diosmin, lapatinib, cyclohexane-1,2,3,4,5,6-hexayl hexakis­(pyridine-3-carboxylate), lactitol, rapamycin, rifampicin, clarithromycin and erythromycin. In contrast to PROTAC compound AZ1, these compounds generally show much greater diversity of solid forms – particularly the large compounds rifampicin, clarithromycin and erythromycin which contain 117–121 atoms compared to 126 in AZ1. These compounds can also be crystallized far more readily than AZ1, for example from cooling of aqueous solutions. The only notable exception to this is lapatinib, where single crystals of the anhydrous freebase form (−341 kJ/mol lattice energy, 66 atoms) could only be produced via an unexpected method using wire as a nucleation device.^55^ Unlike AZ1, however, lapatinib exhibits two anhydrous polymorphs and the commercially available ditosylate salts are highly polymorphic.^56^ This suggests that PROTAC AZ1 stands alone within this subset of structures as being particularly difficult to crystallize yet producing crystal structures with particularly strong lattice energies.

**Figure 8:**
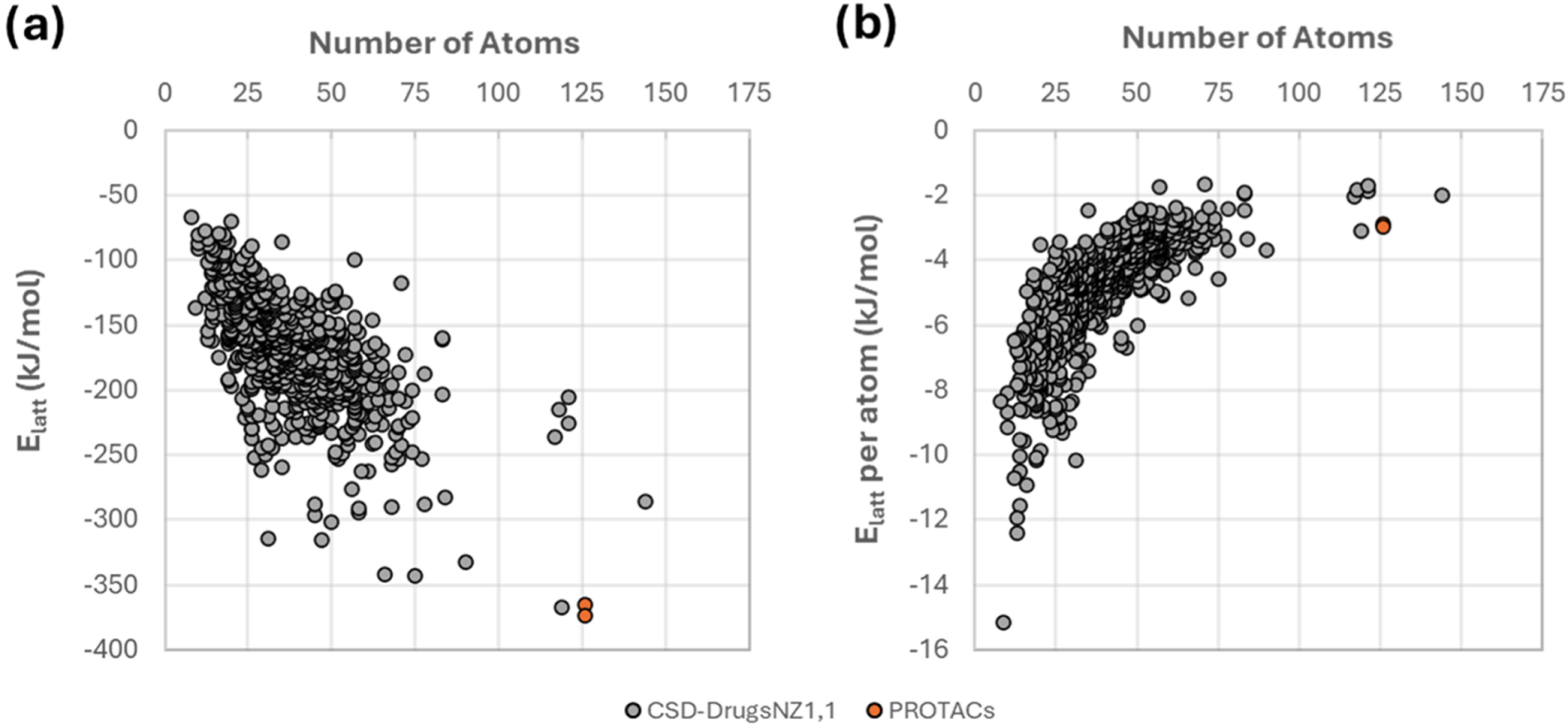
Dependence of the (a) lattice energy (*E*_latt_) and (b) lattice energy per atom with the number of atoms of the crystallizing compound for the CSD-drugs_NZ1,1_ subset (gray), and AZ1 forms 1 and 3 structures reported in this work (orange).

The lattice energies per atom are shown in Figure 8b for the CSD-drugs_NZ1,1_ subset and AZ1 Forms 1 and 3. The lattice energy per atom becomes less stabilizing the larger the drug compound becomes, converging toward −2 kJ/molatom at large compound sizes. Smaller compounds (<25 atoms) crystallize with significantly more stabilizing lattice energies per atom (between −15 to −4 kJ/molatom) than larger compounds. This trend suggests that drug compounds are less able to optimize directional intermolecular interactions such as hydrogen-bonds as they become larger, with a greater lattice energy contribution from dispersion. Since the molecular weight of AZ1 falls roughly in the middle of the average range for PROTAC compounds at 859 g/mol,^57^ it is likely that the low lattice energies per atom observed for AZ1, at −3.0 and −2.9 kJ/molatom for Forms 1 and 3 respectively, are representative of many similar bRo5 compounds. Interestingly, 50% of the crystal structures containing larger compounds (with >69 atoms) contain structural voids which could potentially contain solvent, as observed in Form 3. This observation suggests that the poor ability of the larger compounds to crystallize often results in structures which must include solvent to densely pack. Just like AZ1, three of the large compounds in the CSD-drugs_NZ1,1_ subset, namely probucol (HAXHET and HAXHET01), cabergoline (SUPBEK and SUPBEK03) and difluprednate (IHOZOW01 and IHOZOW02), crystallize in both a close packed structure and one containing structural voids.

### Comparison of AZ1 Bulk Forms

Thermal analysis and solution NMR spectroscopy (SI Figures S8–S9) reveal that Form 1 is anhydrous, with a melt onset at 256 or 262 °C depending on whether obtained from AZ1_mix_ or AZ1_RRS_ respectively. This aligns with the observation that Form 1 containing only AZ1_RRS_ has a slightly more crystalline powder pattern than the sample produced by slurrying AZ1_mix_ in acetonitrile, and the high melting points of both samples are commensurate with the high calculated lattice energy of Form 1. The FTIR spectra for both samples are indistinguishable (SI Figure S10). Thermal and spectral characterization (SI Figures S12–S14) also reveal that Form 2 is a nonstoichiometric solvate form containing dichloromethane, obtained as needles by cooling crystallization. A sample obtained from a cooling crystallization in chloroform appears to be isostructural with Form 2 based on XRPD analysis, thermal data and FTIR data (SI Figures S14–S16). Unlike Form 1, the crystallization of Form 2 was very challenging to reproduce and so only a few tens of milligrams of sample could be generated, mostly derived from AZ1_mix_ which was used for all the following characterization. The slightly humped baseline in the XRPD pattern (Figure 1b) and broad signals in both the FTIR (SI Figure S16) and solid-state NMR spectra (SI Figure S2) of this sample reveal that it is disordered. A miniscule quantity of Form 2 produced using AZ1_RRS_ had an identical XRPD pattern but slightly sharper FTIR features suggesting less disorder. Thermal analysis shows that Form 2 undergoes a gradual solvent loss of roughly 1.3% mass between 25 and 100 °C (0.13 molar eq. of dichloromethane) followed by a sharper solvent loss of 2.4% starting at roughly 150 °C (0.25 molar eq. of dichloromethane), for a combined total stoichiometry of 1:0.38. Given the volatility of the solvent, it is possible that a greater molar ratio of solvent is present in Form 2 before the crystals are filtered and analyzed. A single *T*_g_ at 159 °C is observed by DSC on the second heating of the sample, indicating the collapse of the desolvated crystal structure into an amorphous phase. This is supported by hot-stage polarized optical microscopy (SI Figure S13) which shows the disappearance of all birefringent particles and the needle-like morphology above 170 °C and upon subsequent cooling to 30 °C. The high *T*_g_ of the resulting amorphous phase indicates that molecular mobility is low and the phase is very kinetically stable, likely due to a sum of many weak dispersive interactions between large molecules as observed in the crystalline phases, but perhaps also because the molecules are not constrained to a single conformation as they are in the crystal and can therefore optimize directional interactions such as hydrogen-bonds at a local scale at the expense of long-range ordering. A small sample of Form 2 heated to 150 °C in an oven for 2 h produces a solid with a fully amorphous powder diffraction pattern and a *T*_g_ at 159 °C (SI Figure S17). However, the FTIR spectrum appears not to change compared to the starting material (SI Figure S18). This could be because the desolvated solvate structure is very similar to the crystalline Form 2 structure.

FTIR analysis (Figure 9) shows significant differences in the carbonyl region (1600–1750 cm^–1^) and N–H region (3100–3500 cm^–1^) of AZ1 between Forms 1 and 2. Form 1 contains three carbonyl bands compared to at least four broad bands in Form 2, suggesting that the structures differ in the degree of hydrogen-bonding to the three carbonyl moieties within AZ1. Coupled with the broader and less defined N–H region of Form 2, this suggests that the N–H donors and C=O acceptors in the AZ1 molecules are involved in more and/or stronger hydrogen bonds in the Form 2 structure compared to Form 1, potentially arising from a more favorable crystal conformation that can only pack densely when supported by solvent molecules such as dichloromethane. The FTIR spectra of Forms 1 and 2 differ also by shifts of 5–10 cm^–1^ in at least ten features between 750–1550 cm^–1^. Two-dimensional ^1^H–^13^C FSLG HETCOR NMR analysis (SI Figure S21) reveals no direct evidence for hydrogen-bonding in either sample since these spectra are dominated by intramolecular correlations, but the differences observed in the carbonyl region (above 165 ppm) between the two crystal forms are consistent with the differences in hydrogen-bonding observed via FTIR. The HETCOR analysis of Form 2 shows that the carbonyl peaks produce only a single weak contact at short contact times, most likely corresponding to the intramolecular correlation between the carbonyl carbon atoms and the NH hydrogen atom in the imide moiety of AZ1. This interaction appears stronger at longer contact times (SI Figure S22) with longer distance dipolar interactions also observed in both crystal forms that can be attributed to correlations between the carbonyl carbon atoms and neighboring alkyl hydrogen atoms.

**Figure 9:**
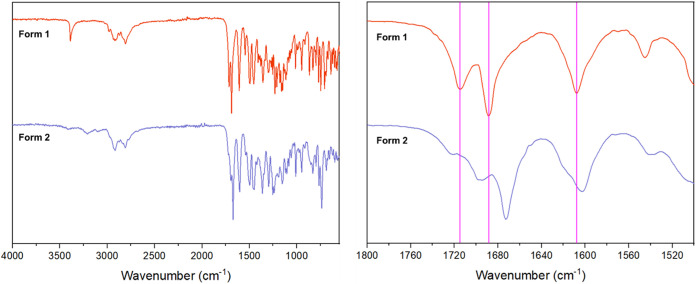
FTIR spectra comparing AZ1 Form 1 and 2. There are differences in the peak breadth and wavenumber in the 1600–1750 and 3100–3500 cm^–1^ regions, suggesting differences in the type and/or degree of hydrogen-bonding.

Since Forms 2 and 3 both present a lamellar-like X-ray powder pattern dominated by a series of strong, evenly spaced peaks that correspond to (00*l*) reflections in the case of Form 3, it is possible that Form 2 possesses a similar channel solvate structure where the main difference is the diameter or spacing of the solvent voids, causing the structural differences along the *c* axis that would cause the (00*l*) reflections to shift uniformly in angle. Applying Bragg’s law to the first four (00*l*) reflections of the Form 3 powder pattern gives an average *d* spacing of 22.6 Å, which is approximately the distance between solvent channels along the *c* axis. Applying Bragg’s law to the first three reflections of the Form 2 powder pattern, and assuming the first peak detected at 1.9° is the (001) reflection, gives a higher *d* spacing of 29.8 Å. This suggests that either the solvent channels are larger in Form 2 than in Form 3, or that the hypothetical AZ1 dimers in Form 2 are more closely aligned with the *c* axis and cause a greater separation of the solvent channels, compared to the somewhat diagonal alignment of AZ1 dimers with the *c* axis observed in Form 3.

The ^13^C NMR spectra (SI Figure S2) for Form 1 samples derived from either AZ1_mix_ or AZ1_RRS_ are very similar and the mixed isomer samples show only slightly more disorder, suggesting that the Form 1 structure is capable of accommodating both diastereomers in the same packing arrangement. The mixed isomer Form 1 crystals may grow as solid solutions like the single crystal of Form 3 analyzed by SC-XRD, giving good diffraction with some disorder from the presence of random isomers. Meanwhile the NMR spectrum of Form 2, derived from AZ1_mix_, is significantly more disordered by comparison and since both NMR and XRPD analysis shows it contains only a single crystalline phase, the disorder likely arises because the Form 2 structure cannot accommodate both isomers as well as Form 1, leading to greater local variation in chemical environments and a more disordered crystal.

The observation that AZ1 has a propensity to form solvates is commensurate with the findings from the CSD analysis that bRo5 compounds are less able to pack densely as pure solids compared to smaller drug molecules, and the available thermal, spectral and diffraction data suggests that the Form 2 structure may be a similar channel solvate to Form 3 but with a greater importance of hydrogen-bonding interactions. The difficulty in drawing conclusions from the limited data on Form 2 illustrates the challenge faced by the pharmaceutical industry in identifying all solid forms of PROTACs. Solvate forms are another challenge for drug development since many solvents used in synthesis are toxic to humans, and because their desolvation behavior can raise challenges regarding their stability, solubility and mechanical properties. While crystallization experiments of AZ1_mix_ that produced Form 1 and Form 2 were reproducible using AZ1_RRS_ and with similar crystallinity, attempts to crystallize pure AZ1_RRR_ consistently produced amorphous solids. This indicates that AZ1_RRR_ is prone to amorphization in the absence of AZ1_RRS_, yet in AZ1_mix_ it appears capable of forming crystalline solid solutions with AZ1_RRS_ in at least Forms 1 and 3 based on spectroscopic and crystallographic data. These findings also suggest that neither stereoisomer impedes the crystal growth of the other.

### Amorphous Solids

Amorphous phases of AZ1 can be produced easily, since the compound is poorly crystallizable. Grinding any AZ1 sample in a ball mill at 20 Hz for as little as 5 min results in an amorphous powder by XRPD analysis, and thermal analysis of the amorphous as-synthesized AZ1_RRR_ material as well as milled samples of AZ1_mix_ and AZ1_RRS_ all show a *T*_g_ value around 157 – 161 °C (SI Figures S23–S25) and broadening of spectral bands in the FTIR spectrum, resembling the amorphous desolvated solvate produced by heating Form 2 to remove the dichloromethane. The high *T*_g_ values suggest that all of these amorphous phases are likely to be very stable with respect to recrystallization at room temperature, forming robust glasses. The milled AZ1_RRS_ solid appears to differ from the others slightly in the sharpness and position of its carbonyl bands between 1680 and 1720 cm^–1^ and between 1600 and 1620 cm^–1^ (Figure 10), potentially indicating differences in the degree of hydrogen bonding to the carbonyl groups within its local structure. The desolvated Form 2 solid also has a slightly sharper feature at 1672 cm^–1^ compared to 1684 cm^–1^ for the others, and two missing bands at 1175 and 867 cm^–1^ suggesting similar local structure differences may be present in this phase. It is possible that greater free volume and fewer conformational restrictions in amorphous form allow AZ1 molecules to form more and/or stronger hydrogen-bonding interactions, broadening the C=O and N–H regions of the FTIR spectra compared to the crystalline phases and resulting in highly stable glasses characterized by a high *T*_g_.

**Figure 10:**
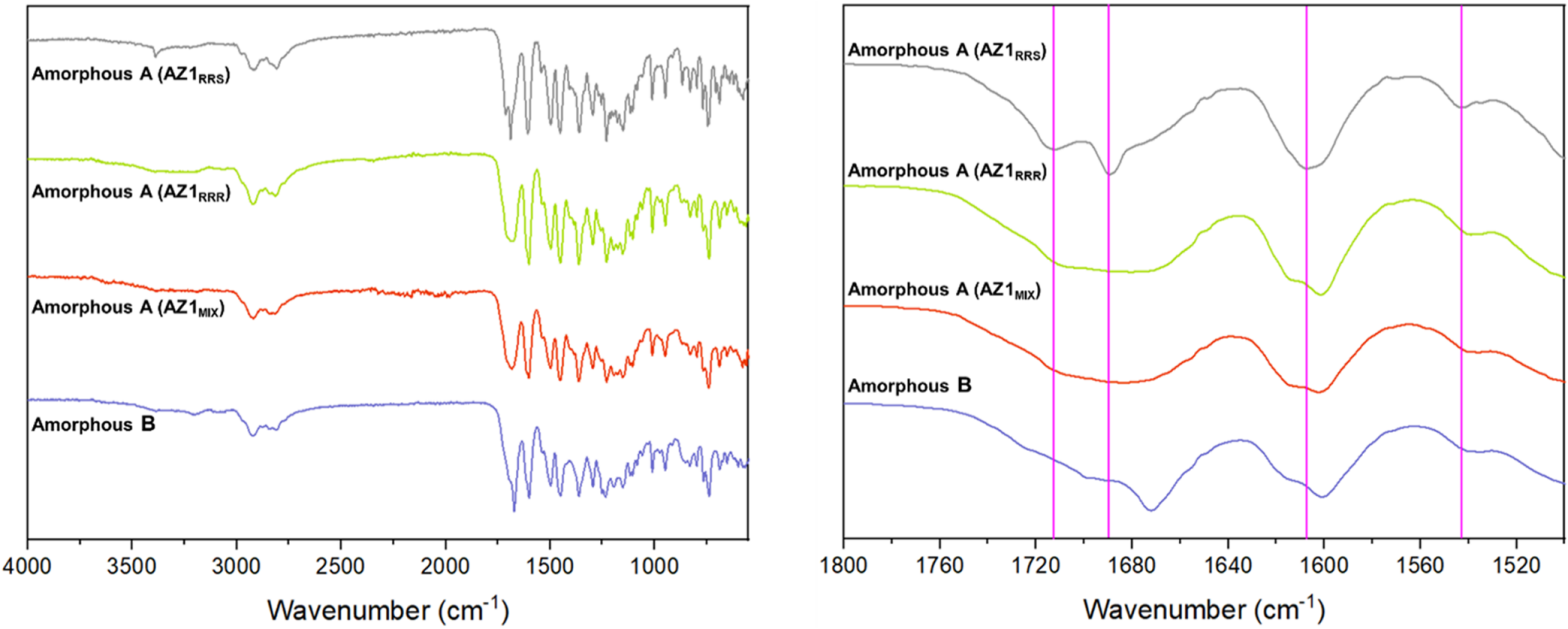
FTIR spectra of AZ1 amorphized by grinding Form 1 in a ball mill (amorphous form A) or by desolvating Form 2 (amorphous form B).

While these spectral differences appear to be relatively minor, a comparison of the dissolution profiles for the amorphous solids produced by milling the anhydrous Form 1 (amorphous form A) and those produced by desolvating the Form 2 solvate (amorphous form B) reveal a much more significant 4-fold difference in apparent solubility. Figure 11a shows nonsink dissolution profiles over 2 h in fasted state simulated intestinal fluid (FaSSIF) at 37 °C, comparing amorphous forms A and B to crystalline Form 1, with all samples produced using AZ1_mix_. To reduce the impact of particle size differences between the samples produced by ball milling or otherwise, the unmilled samples were ground gently in a pestle and mortar and all powders were sieved to remove particles larger than 150 μm. SEM images (Figure 12) confirm that all samples consisted of particles in the range of 1–10 μm in length with similar dispersity and show a consistent particle morphology. Despite this, amorphous form A showed a considerable apparent solubility increase compared to the crystalline Form 1 whereas amorphous form B showed a far lower solubility, almost matching the crystalline Form 1. Since the FTIR comparison of crystalline Forms 1 and 2 appears to indicate more and/or stronger hydrogen-bonds in the latter form, it is possible that amorphous form B also contains more and/or stronger hydrogen-bonds than amorphous form A, that stabilize it against dissolution. This greater stabilization of the amorphous phase via molecular interactions is not evident from thermal analysis however, with all amorphous solids presenting a very similar *T*_g_. This demonstrates that while amorphous forms of AZ1 prepared by different methods may appear similar by standard characterization techniques, potential differences in their local structure cause their dissolution behavior to vary significantly.

**Figure 11:**
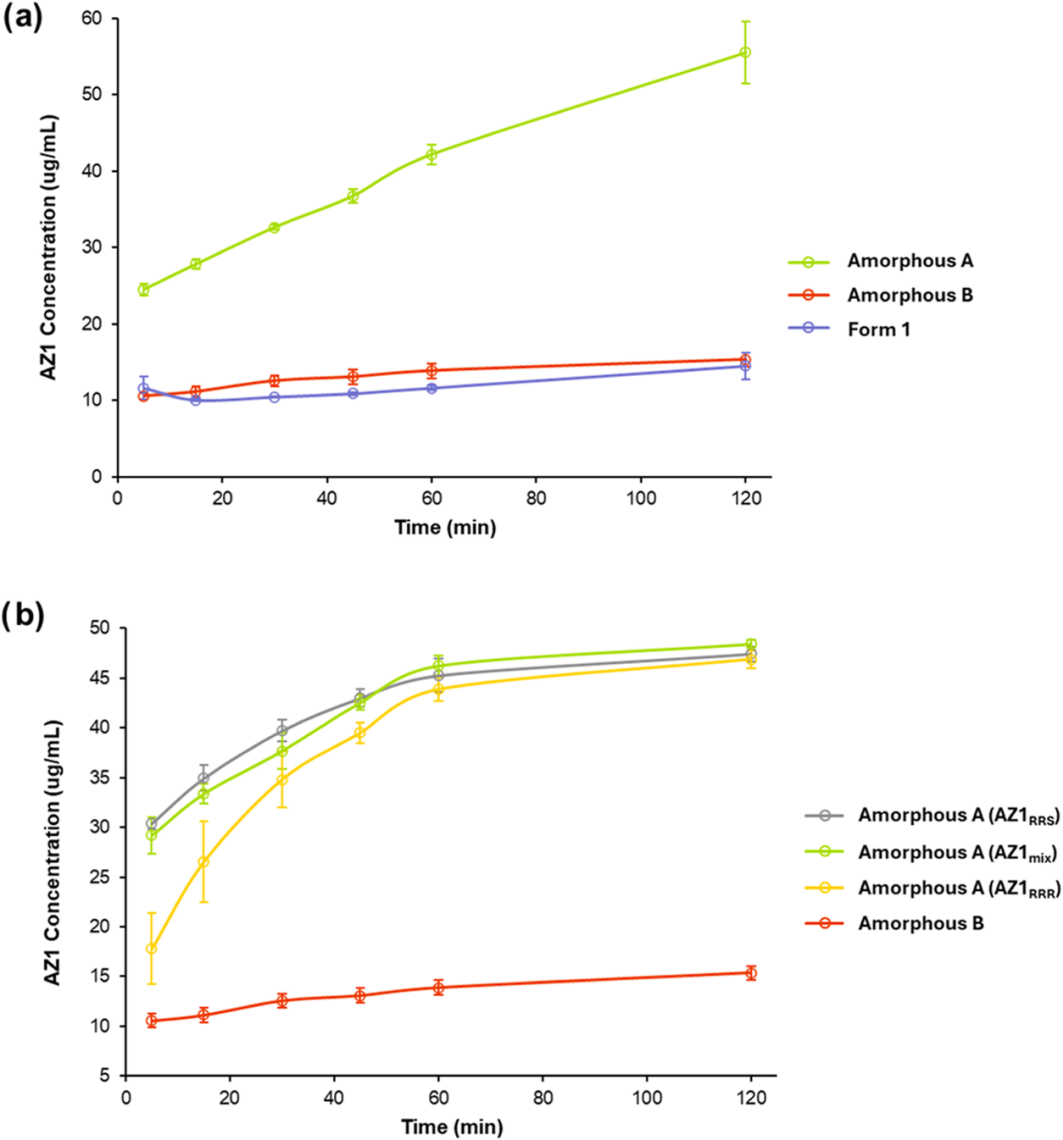
Nonsink dissolution profiles over 2 h in fasted state simulated intestinal fluid (FaSSIF) at 37 °C, using a 10-fold nonsink condition relative to the crystalline solubility (Form 1). (a) Dissolution profiles of AZ1 Form 1 crystals, amorphous form A and amorphous form B, with all three samples prepared using AZ1_mix_. (b) Dissolution profiles of amorphous form A samples produced separately from AZ1_mix_, AZ1_RRR_ and AZ1_RRS_ as well as amorphous form B. AZ1_mix_ and AZ1_RRS_ were milled to produce amorphous form A while AZ1_RRR_ was used as synthesized without milling. Average concentrations and error bars are shown for time-points acquired in triplicate for both plots.

**Figure 12:**
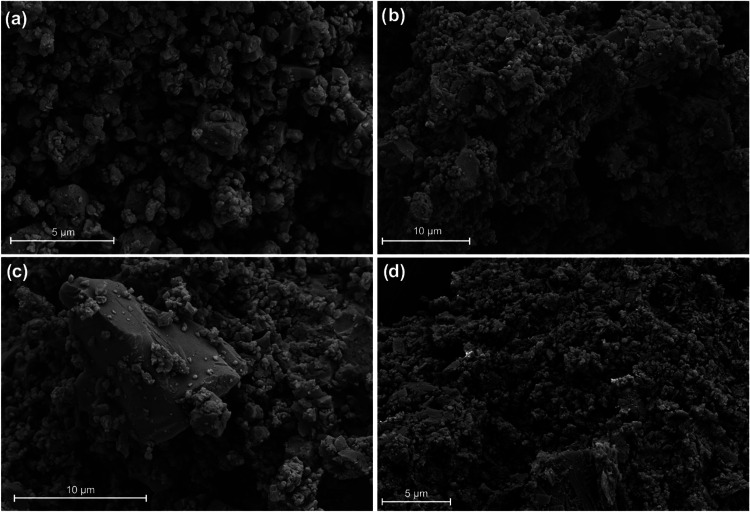
SEM images of (a) AZ1_MIX_ ball-milled, (b) AZ1_RRS_ ball-milled, (c) AZ1_RRR_ as synthesized and (d) desolvated Form 2. While the milled samples are very similar with most particles between 1–5 μm in length, AZ1_RRR_ shows greater polydispersity with the presence of larger particles above 10 μm.

The dissolution profiles of amorphous form A produced by ball-milling AZ1_mix_ or AZ1_RRS_ were compared to the as-synthesized amorphous AZ1_RRR_ (unmilled) and amorphous form B (Figure 11b). Again, the unmilled AZ1_RRR_ samples were ground gently by hand and sieved to partially control for particle size differences. The two milled and the unmilled AZ1_RRR_ samples of amorphous form A have a similar dissolution profile reaching approximately the same apparent solubility, with the initially slower dissolution of AZ1_RRR_ likely arising from a more polydisperse particle size since it was not ball-milled. Indeed, SEM images (Figure 12c) show that this sample contains some larger particles. This may also explain the greater variation between individual repeats in the early time-points. After 30 min of dissolution, the AZ1_RRR_ sample began to overlap with the dissolution profiles of the other two amorphous form A solids and the independent repeats become much more similar. Again, amorphous form B has a much flatter dissolution profile and much lower solubility than all the others. This behavior may well be an example of *pseudo*-polyamorphism in which different amorphous forms, prepared in different ways, exhibit different physical properties without an observed first order phase transition in between them.^58^ This type of phenomenon has been observed for the antibiotic roxithromycin (four different amorphous forms distinguishable by their particle morphology, thermodynamics and dissolution behavior^59^), simvastatin (cryo-milling and quench-cooling of the melt^60^), the diuretic hydrochlorothiazide (three distinct amorphous forms prepared by spray-drying, quench-cooling and ball milling^61^) and the antihypertensive drug valsartan (two amorphous forms distinguished by solid-state NMR and dissolution tests^62^). In contrast, amorphous celecoxib prepared in different ways gives materials exhibiting similar physicochemical properties.^63^ The existence of polyamorphism represents both a challenge and an interesting opportunity in pharmaceutical intellectual property and further highlights the complexity of the solid forms landscape of PROTACs.^64^

## Conclusions

PROTAC compounds such as AZ1 are poorly crystallizable and their slow crystal growth kinetics hinder the discovery of crystalline forms, even by high-throughput crystallization techniques. When AZ1 crystals were obtained by larger scale crystallization techniques, only one crystal was suitable for structural analysis using synchrotron-source X-ray diffraction and another microcrystalline powder was suitable only for analysis using electron diffraction. These analyses revealed two AZ1 crystal structures built almost entirely on dispersive interactions such as aliphatic stacking with very low importance of hydrogen-bonding, and with similarly elongated crystal conformations to maximize surface area for dispersive interactions. The generally poor crystallizability and slow growth kinetics of AZ1 can be explained by the lack of strong, directional interactions present and the reliance instead on the sum of many weak, nondirectional interactions. AZ1 Forms 1 and 3 have two of the highest lattice energies in the CSD-drugs subset yet have among the lowest lattice energy per atom in the same subset of structures, reflecting their inability to optimize strong, directional interactions and explaining why they have poor solubility and high melting points. Like similarly large drugs in the subset, AZ1 crystallizes in two solvate forms of which at least one is a channel solvate containing voids, exemplifying the poor ability of bRo5 compounds to densely pack in the crystalline phase. Thermal and spectral characterization suggest that Form 2 may have a similar channel solvate structure to Form 3, and that not all forms are equally capable of accommodating different stereoisomers. AZ1 may also exhibit *pseudo*-polyamorphism, with amorphous solids produced by desolvating Form 2 crystals displaying very different dissolution characteristics to those produced by milling the anhydrous Form 1 crystals. This study demonstrates how standard solid-form screening approaches for small-molecule drugs may be insufficient for more complex bRo5 compounds such as PROTACs. Given their crystallization challenges, the pharmaceutical industry may need to recalibrate its expectations around the time and effort required to explore these compounds’ solid form landscapes, and more advanced methods that combine the efficiency of high-throughput screening with the control of conventional crystallization may be essential for developing PROTAC drug products.

## Supplementary Material



## Notes

Full ENaCt experiment details, additional crystallographic data for AZ1 Form 1 and Form 3, solid-state NMR spectra, full interaction maps of Forms 1 and 3, additional XRPD patterns, additional CrysIn interaction pairs, additional thermal analysis, HS-POM analysis and FTIR spectra (PDF)

## Notes

The underlying data files are available free of charge at DOI: 10.15128/r1xg94hp618, and the underlying 3D ED data is available at DOI: 10.5281/zenodo.15355560.

## Abbreviations

bRo5: beyond-rule-of-5
API: active pharmaceutical ingredient
TPD: targeted protein degradation
PROTAC: proteolysis targeting chimera
CRBN: cereblon
HBA: hydrogen-bond acceptor
HBD: hydrogen-bond donor
ER: estrogen receptor
XRPD: X-ray powder diffraction
LAG: liquid-assisted grinding
ENaCt: encapsulated nanodroplet crystallization
3D ED: 3-dimensional electron diffraction
SC-XRD: single crystal X-ray diffraction
SEM: scanning electron microscopy
CSD: Cambridge structural database
OCC: open computational chemistry
DSC: differential scanning calorimetry
TGA: thermogravimetric analysis
SSNMR: solid-state nuclear magnetic resonance
HS-POM: hot-stage polarized optical microscopy
HETCOR: heteronuclear correlation
UPLC: ultra performance liquid chromatography
FaSSIF: fasted state simulated intestinal fluid
DOSY: diffusion ordered spectroscopy
DFT: density functional theory
