# Supplementary material for: Crystallizing the Uncrystallizable: Insights from Extensive Screening of PROTACs
Source: J Am Chem Soc. 2025 Jul 23;147(31):28056–72. doi: 10.1021/jacs.5c07977 (PMC12333357; doi:10.1021/jacs.5c07977)
Supplement: Supplementary file 1 [file ja5c07977_si_001.pdf]

SUPPLEMENTARY INFORMATION

Crystallizing the Un-crystallizable: Insights from Extensive Screening of PROTACs

Martin A. Screen,[1] James F. McCabe,[2] Sean Askin,[3] Jamie L. Guest,[1] Paul Hodgkinson,[1] Aurora J. Cruz-Cabeza,[1] Toby J. Blundell,[1] Daniel N. Rainer,[4] Simon J. Coles,[4] Alexandra Longcake,[5] Michael R. Probert,[5] Clare S. Mahon,[1] Mark R. Wilson,[1] Jonathan W. Steed[1*]

[1]Durham University, Department of Chemistry, South Road, Durham DH1 3LE, United Kingdom. [2]Early Pharmaceutical Development & Manufacture, Pharmaceutical Sciences, R&D, AstraZeneca, Macclesfield SK10 2NA, United Kingdom. [3]Advanced Drug Delivery, Pharmaceutical Sciences, R&D, AstraZeneca, Cambridge CB2 0AA, United Kingdom. [4]School of Chemistry and Chemical Engineering, University of Southampton, Highfield Campus, Southampton, SO17 1BJ, United Kingdom. [5]School of Natural and Environmental Sciences, Bedson Building, Newcastle University, Kings Road, NE1 7RU, United Kingdom.

Table 1. Approximate solubility data for $AZ1_{mix}$ assessed during crystallisation screening. The solubility of AZ1 was first assessed at 10 mg/mL in each solvent. If soluble without heating, the concentration was doubled by adding more AZ1 powder until heat was required to fully dissolve the powder. Solvents in which AZ1 appeared partially soluble at 10 mg/mL were halved in concentration by addition of more solvent until the powder could be fully dissolved with heating. Solvents in which AZ1 appeared completely insoluble at 10 mg/mL were not studied further.

Solvent	Solubility (mg/mL)	Solvent	Solubility (mg/mL)	Solvent	Solubility (mg/mL)
1,2-dibromoethane	10	Benzyl alcohol	5	Ethyl acetate	<1
1,2,4-trichlorobenzene	<1	Chlorobenzene	10	Hexane	<1
1,4-dioxane	62	Chloroform	114	Isopropanol	<1
1-butanol	5	Cyclohexanone	10	Mesitylene	<1
1-pentanol	5	Cyclopentanone	120	Methanol	<1
1-propanol	<1	Diethyl ether	<1	Nitrobenzene	10
2-butanol	3.3	Diethylene glycol	10	Nitromethane	5
2-butanone	5	Diisopropyl ether	<1	Pyridine	224
2-picoline	158	Dichloromethane	112	Tetrahydrofuran	170
4-ethyl pyridine	188	Dimethylacetamide	200	Toluene	<1
Acetonitrile	1.25	Dimethylformamide	84	Water	<1
Acetone	<1	Dimethylsulfoxide	104	p-xylene	<1
Benzene	<1	Ethanol	2.5		

Table 2. ENaCt plate layouts for $AZ1_{mix}$ crystallisation experiments.

**Plate 1 layout:**		**200 nL of oils**											
**50 nL of AZ1 solution (approximate concentration/ mg mL^{-1})**		**1**	**2**	**3**	**4**	**5**	**6**	**7**	**8**	**9**	**10**	**11**	**12**
DMSO (5.2 mg mL^{-1})	**A**	No oil	PDMSO					No oil	Fomblin Y				
DMSO (5.2 mg mL^{-1})	**B**	No oil	FC-40					No oil	Mineral oil				
DMF (41.7 mg mL^{-1})	**C**	No oil	PDMSO					No oil	Fomblin Y				
DMF (41.7 mg mL^{-1})	**D**	No oil	FC-40					No oil	Mineral oil				
DCM (10.4 mg mL^{-1})	**E**	No oil	PDMSO					No oil	Fomblin Y				
DCM (10.4 mg mL^{-1})	**F**	No oil	FC-40					No oil	Mineral oil				
DESO (20.8 mg mL^{-1})	**G**	No oil	PDMSO					No oil	Fomblin Y				
DESO (20.8 mg mL^{-1})	**H**	No oil	FC-40					No oil	Mineral oil				
**Plate 2 layout:**		**200 nL of oils**											
**50 nL of AZ1 solution (approximate concentration/ mg mL^{-1})**		**1**	**2**	**3**	**4**	**5**	**6**	**7**	**8**	**9**	**10**	**11**	**12**
Toluene (5.2 mg mL^{-1})	**A**	No oil	PDMSO					No oil	Fomblin Y				
Toluene (5.2 mg mL^{-1})	**B**	No oil	FC-40					No oil	Mineral oil				
DCE (5.2 mg mL^{-1})	**C**	No oil	PDMSO					No oil	Fomblin Y				
DCE (5.2 mg mL^{-1})	**D**	No oil	FC-40					No oil	Mineral oil				
2-Me THF (5.2 mg mL^{-1})	**E**	No oil	PDMSO					No oil	Fomblin Y				
2-Me THF (5.2 mg mL^{-1})	**F**	No oil	FC-40					No oil	Mineral oil				
THTP-1-oxide (20.8 mg mL^{-1})	**G**	No oil	PDMSO					No oil	Fomblin Y				
THTP-1-oxide (20.8 mg mL^{-1})	**H**	No oil	FC-40					No oil	Mineral oil				
**Plate 3 layout:**		**200 nL of oils**											
**50 nL of AZ1 solution (approximate concentration/ mg mL^{-1})**		**1**	**2**	**3**	**4**	**5**	**6**	**7**	**8**	**9**	**10**	**11**	**12**
EtOAc (5.2 mg mL^{-1})	**A**	No oil	PDMSO					No oil	Fomblin Y				
EtOAc (5.2 mg mL^{-1})	**B**	No oil	FC-40					No oil	Mineral oil				
MeCN (5.2 mg mL^{-1})	**C**	No oil	PDMSO					No oil	Fomblin Y				
MeCN (5.2 mg mL^{-1})	**D**	No oil	FC-40					No oil	Mineral oil				
4-Methyl-2-pentanone (5.2 mg mL^{-1})	**E**	No oil	PDMSO					No oil	Fomblin Y				
4-Methyl-2-pentanone (5.2 mg mL^{-1})	**F**	No oil	FC-40					No oil	Mineral oil				
Nitromethane (5.2 mg mL^{-1})	**G**	No oil	PDMSO					No oil	Fomblin Y				
Nitromethane (5.2 mg mL^{-1})	**H**	No oil	FC-40					No oil	Mineral oil				
**Plate 4 layout:**		**200 nL of oils**											
**50 nL of AZ1 solution (approximate concentration/ mg mL^{-1})**		**1**	**2**	**3**	**4**	**5**	**6**	**7**	**8**	**9**	**10**	**11**	**12**
DMAC (20.8 mg mL^{-1})	**A**	No oil	PDMSO					No oil	Fomblin Y				
DMAC (20.8 mg mL^{-1})	**B**	No oil	FC-40					No oil	Mineral oil				
1,4-dioxane (5.2 mg mL^{-1})	**C**	No oil	PDMSO					No oil	Fomblin Y				
1,4-dioxane (5.2 mg mL^{-1})	**D**	No oil	FC-40					No oil	Mineral oil				
NMI (5.2 mg mL^{-1})	**E**	No oil	PDMSO					No oil	Fomblin Y				
NMI (5.2 mg mL^{-1})	**F**	No oil	FC-40					No oil	Mineral oil				
piperidine (5.2 mg mL^{-1})	**G**	No oil	PDMSO					No oil	Fomblin Y				
piperidine (5.2 mg mL^{-1})	**H**	No oil	FC-40					No oil	Mineral oil				

Table 3. ENaCt results from plate readouts. F – fail; 1 – oil only or still solvated; 2 – amorphous or non-crystalline material; 3 – small single crystals or microcrystalline material.

**Plate 1 results**	**1**	**2**	**3**	**4**	**5**	**6**	**7**	**8**	**9**	**10**	**11**	**12**
**A**	1	2	2	2	2	1	2	1	1	1	1	1
**B**	1	2	2	2	2	2	2	1	1	1	1	1
**C**	1	2	2	2	2	2	2	1	1	1	1	1
**D**	1	2	2	2	2	2	2	2	1	1	1	1
**E**	1	2	2	2	2	2	1	1	1	1	1	1
**F**	1	1	2	2	2	2	1	1	2	2	2	2
**G**	1	1	F	1	1	1	1	1	1	1	1	1
**H**	1	1	F	1	1	1	1	3	1	1	1	1
**Plate 2 results**	**1**	**2**	**3**	**4**	**5**	**6**	**7**	**8**	**9**	**10**	**11**	**12**
**A**	2	2	2	2	2	2	2	2	2	2	2	2
**B**	2	2	2	2	2	2	2	1	1	1	2	2
**C**	1	2	1	1	1	1	1	1	1	1	1	1
**D**	1	1	1	1	1	1	1	1	1	1	1	1
**E**	1	1	1	1	1	1	1	1	1	1	1	1
**F**	1	1	1	1	1	1	1	1	1	1	1	1
**G**	1	1	1	1	1	1	1	1	1	1	1	1
**H**	1	1	1	1	1	1	1	1	1	1	1	1
**Plate 3 results**	**1**	**2**	**3**	**4**	**5**	**6**	**7**	**8**	**9**	**10**	**11**	**12**
**A**	1	1	1	1	1	1	2	2	2	1	1	1
**B**	2	2	2	2	2	2	2	1	1	1	1	2
**C**	2	2	2	2	2	2	2	2	2	2	2	2
**D**	2	2	2	2	2	2	2	2	2	2	2	2
**E**	1	1	1	1	1	1	1	1	1	1	1	1
**F**	1	1	1	1	1	1	1	1	1	1	1	1
**G**	2	2	2	2	2	2	2	2	2	2	2	2
**H**	2	2	2	2	2	2	2	2	2	2	2	2
**Plate 4 results**	**1**	**2**	**3**	**4**	**5**	**6**	**7**	**8**	**9**	**10**	**11**	**12**
**A**	2	1	1	1	1	1	2	1	1	1	1	1
**B**	2	1	1	1	1	2	2	1	1	1	1	1
**C**	1	1	1	1	1	1	1	F	1	1	1	1
**D**	1	1	1	1	1	1	1	F	1	1	1	1
**E**	2	2	2	2	2	2	2	1	2	2	1	1
**F**	1	1	1	1	1	1	1	1	1	1	1	1
**G**	1	1	1	1	1	1	2	1	2	3	1	1
**H**	1	2	2	2	1	2	2	1	1	1	1	1

Table 4. Crystallographic data for AZ1 Form 1.

**Empirical formula**	$C_{49}H_{63}FN_{10}O_3$	**μ/mm^{-1}**	n/a
**Formula weight**	859.108	**F(000)**	744.1
**Temperature/K**	175(5)	**Crystal size /μm^3**	1.8 × 0.65 × n/a
**Crystal system**	monoclinic	**Radiation**	electron (λ = 0.0251 Å)
**Space group**	*I*2	**2Θ range for data collection/°**	0.16 to 1.8
**a/ Å**	9.4516(8)	**Index ranges**	-11 ≤ h ≤ 11, -7 ≤ k ≤ 7, -85 ≤ l ≤ 85
**b/ Å**	6.2776(4)	**Reflections collected**	10286
**c/ Å**	75.233(16)	**Independent reflections**	10284
**α/°**	90	**Data/ restraints/ parameters**	10284/706/571
**β/°**	92.476(10)	**Goodness-of-fit on F^2**	1.177
**γ/°**	90	**Final R indexes [I >=2σ (I)]**	R_1 = 0.1539, wR_2 = 0.3464
**Volume/Å^3**	4459.7(10)	**Final R indexes (all data)**	R_1 = 0.2134, wR_2 = 0.3828
**Z**	4	**Largest diff. peak /hole (Olex2 N-beam)**	1.03/-1.16
**ρ_{calc} g/cm^3**	1.280	**Z-score (Olex2)**	17.40 (9.19 raw)

Table 5. Hydrogen bond for AZ1 Form 1.

D	H	A	d(D-H)/Å	d(H-A)/Å	d(D-A)/Å	D-H-A/°
N9	H9	O1	1.027	2.262	3.269(16)	166.2(4)

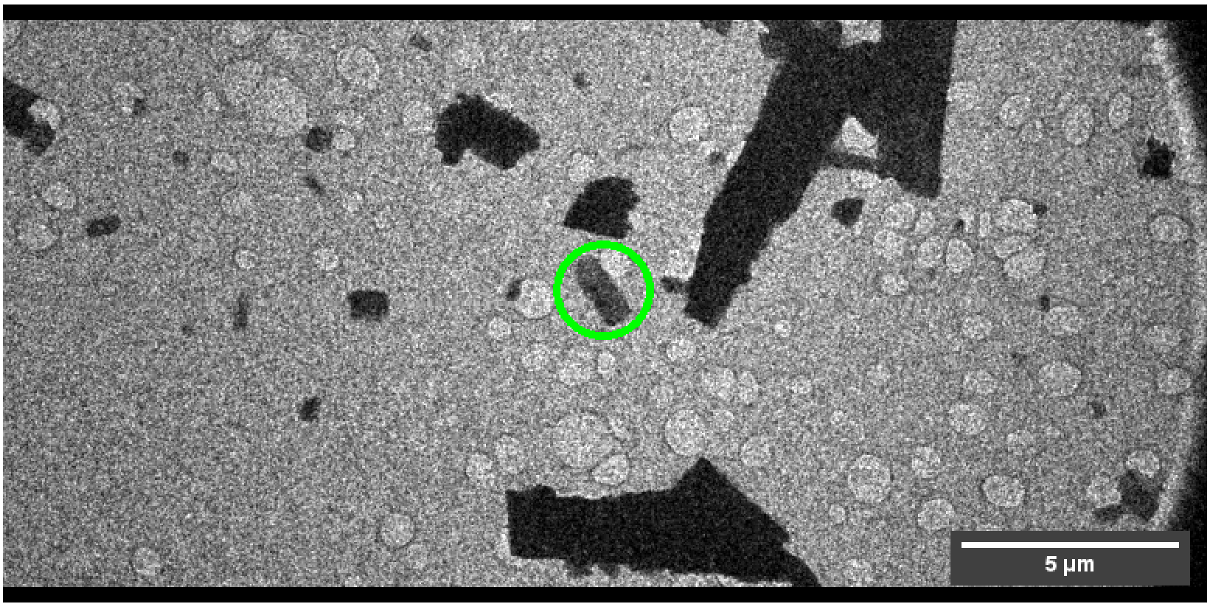

Figure **S1**. Image of crystal used for 3D ED structure determination, the green circle indicating the size and position of the selected area aperture.

Table 6. Crystallographic data for AZ1 Form 3.

**Empirical formula**	$C_{55}H_{70.5}FN_{10}O_3$	**μ/mm^{-1}**	0.226
**Formula weight**	938.71	**F(000)**	1007.0
**Temperature/K**	100.00	**Crystal size /mm^3**	0.089 × 0.008 × 0.002
**Crystal system**	monoclinic	**Radiation**	Synchrotron (λ = 1.0402)
**Space group**	$P2_1$	**2Θ range for data collection/°**	1.786 to 79.728
**a/ Å**	12.0183(5)	**Index ranges**	-14 ≤ h ≤ 14, -7 ≤ k ≤ 7, -40 ≤ l ≤ 41
**b/ Å**	6.2727(2)	**Reflections collected**	18586
**c/ Å**	33.8422(11)	**Independent reflections**	8942 [R_{int} = 0.0659, R_{sigma} = 0.1137]
**α/°**	90	**Data/ restraints/ parameters**	8942/1377/666
**β/°**	99.389(3)	**Goodness-of-fit on F^2**	0.957
**γ/°**	90	**Final R indexes [I >=2σ (I)]**	R_1 = 0.0492, wR_2 = 0.1094
**Volume/Å^3**	2517.09(16)	**Final R indexes (all data)**	R_1 = 0.0823, wR_2 = 0.1188

Z	2	Largest diff. peak /hole / e Å^{-3}	0.19/-0.25
ρ_{calc} g/cm^3	1.239	Flack parameter	0.4(4)

Table 7. Hydrogen bond for AZ1 Form 3.

D	H	A	d(D-H)/Å	d(H-A)/Å	d(D-A)/Å	D-H-A/°
N9	H9	O1A^2	0.88	1.98	2.628(19)	129.7

^{1}+X,1+Y,+Z; ^{2}1-X,-1/2+Y,1-Z

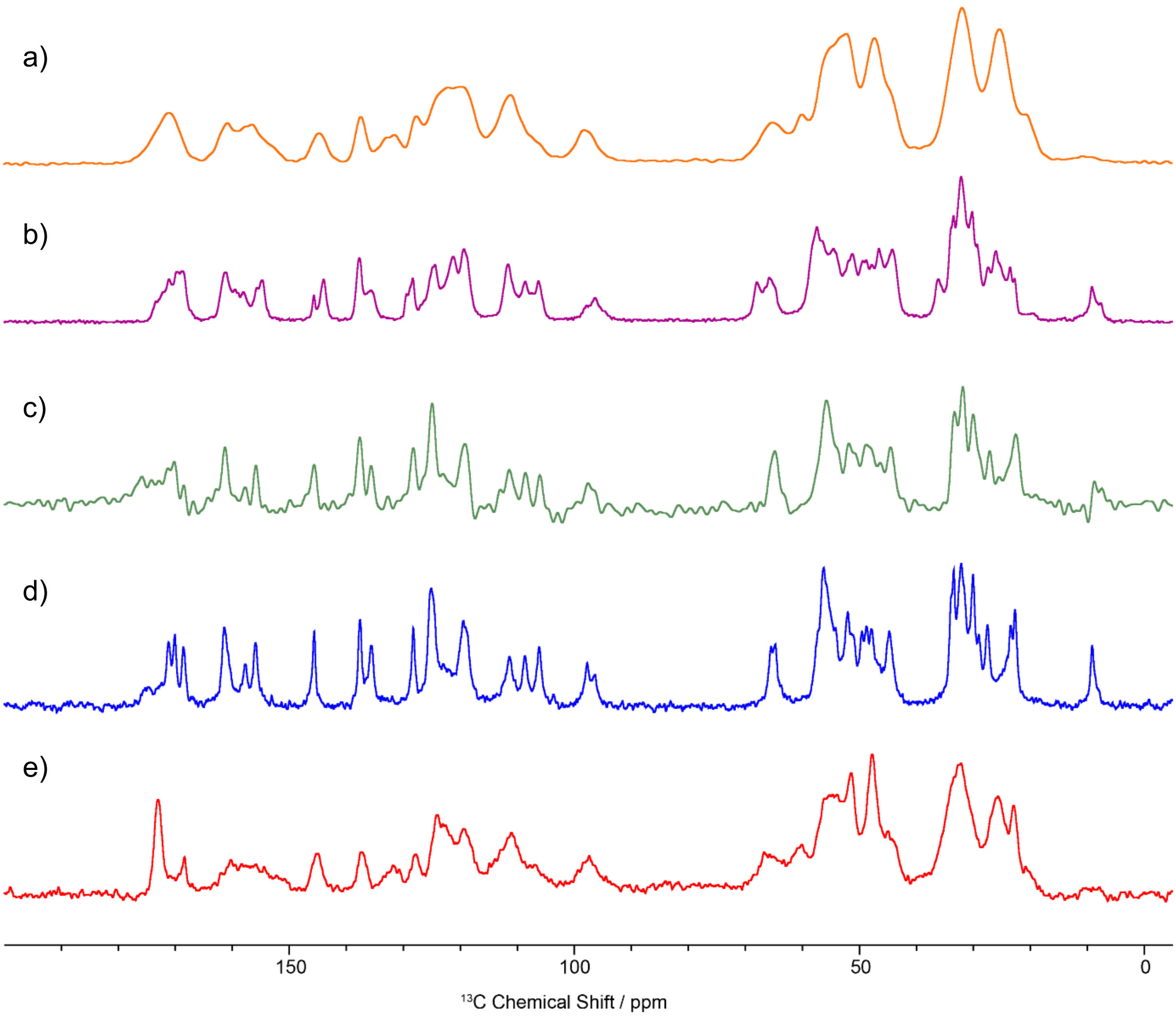

Figure **S2**. Solid-state ^{13}C NMR spectra of AZ1 samples. a) Ball milled AZ1$_{mix}$ (amorphous type A), b) AZ1$_{mix}$ as-synthesised (Form 1), c) AZ1$_{mix}$ slurried in MeCN (Form 1), d) AZ1$_{RRS}$ as-synthesised (Form 1) and e) AZ1$_{mix}$ Form 2. NMR analysis of other AZ1 samples was prevented by limited sample quantities. Spectrum c) was acquired with a relatively low sample quantity.

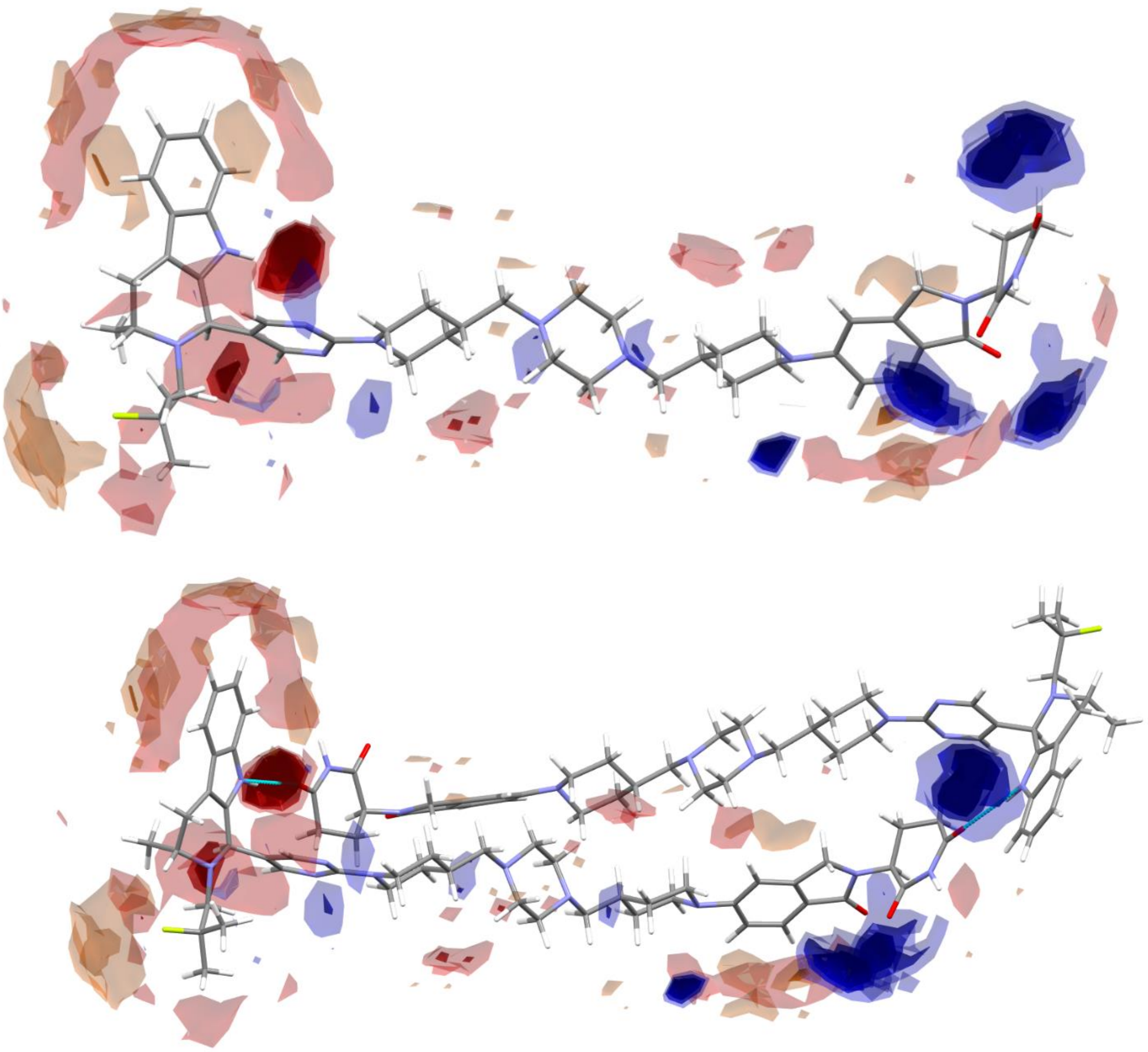

Figure **S3**. Full interaction maps of AZ1 Form 1 calculated using Mercury.[50] Red surfaces indicate a predicted probability of hydrogen-bond acceptors; blue surfaces indicate a predicted probability of hydrogen-bond donors; brown surfaces indicate a hydrophobic preference.

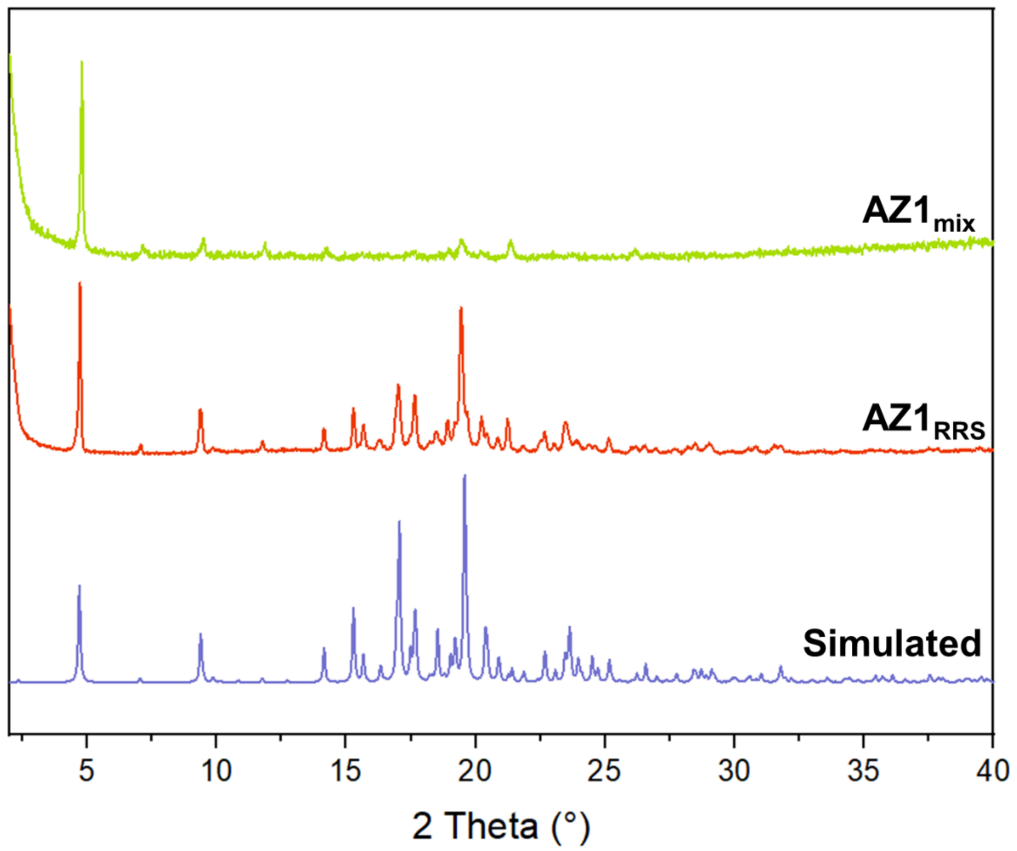

Figure **S4**. XRPD patterns for Form 1 produced using $AZ1_{mix}$ (top) and $AZ1_{RRS}$ (centre) compared to the simulated pattern from single crystal data (bottom), confirming phase purity of the bulk Form 1 samples.

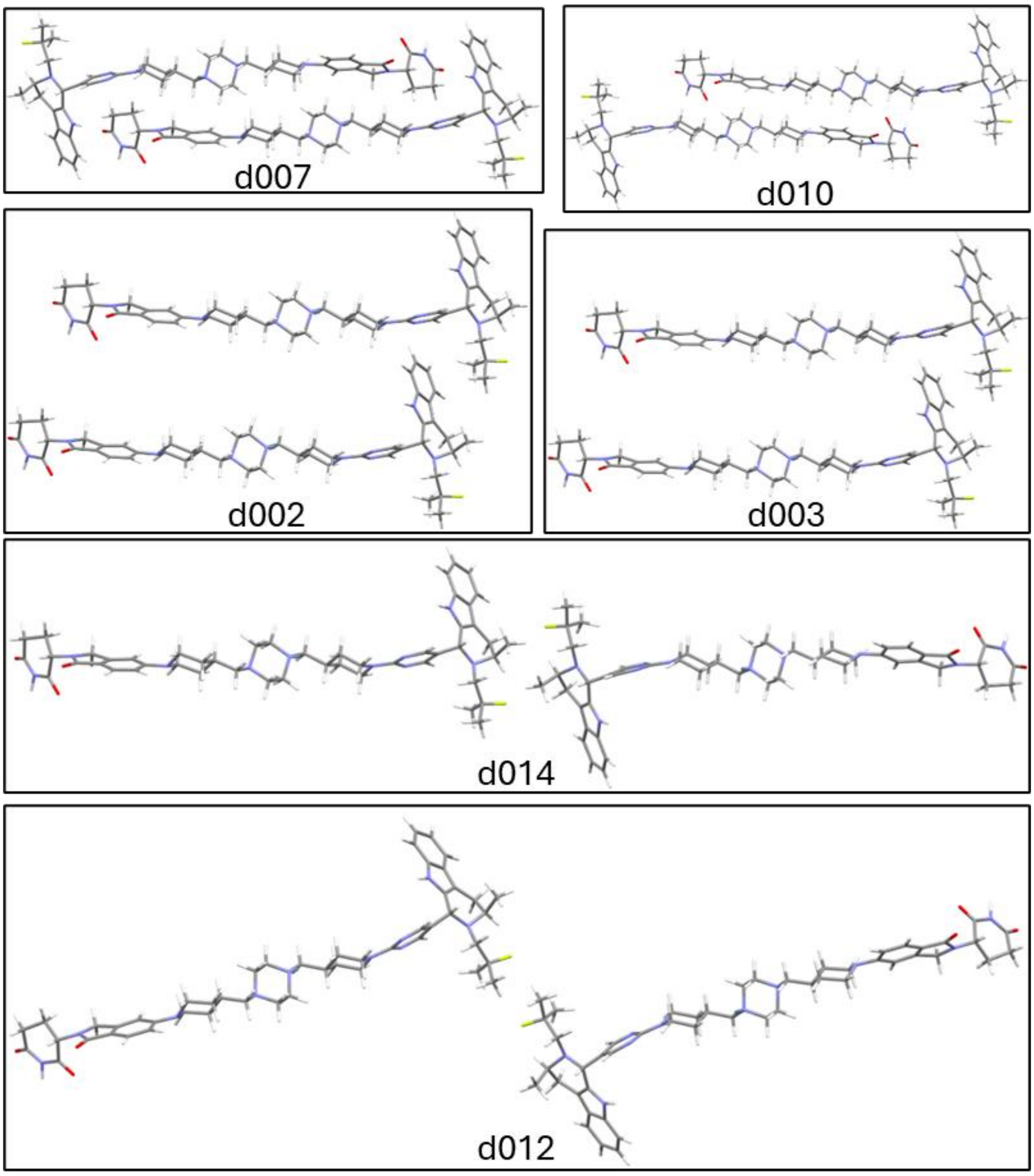

Figure **S5**. CrysIn interaction pairs for Form 1 not included in Figure 6.

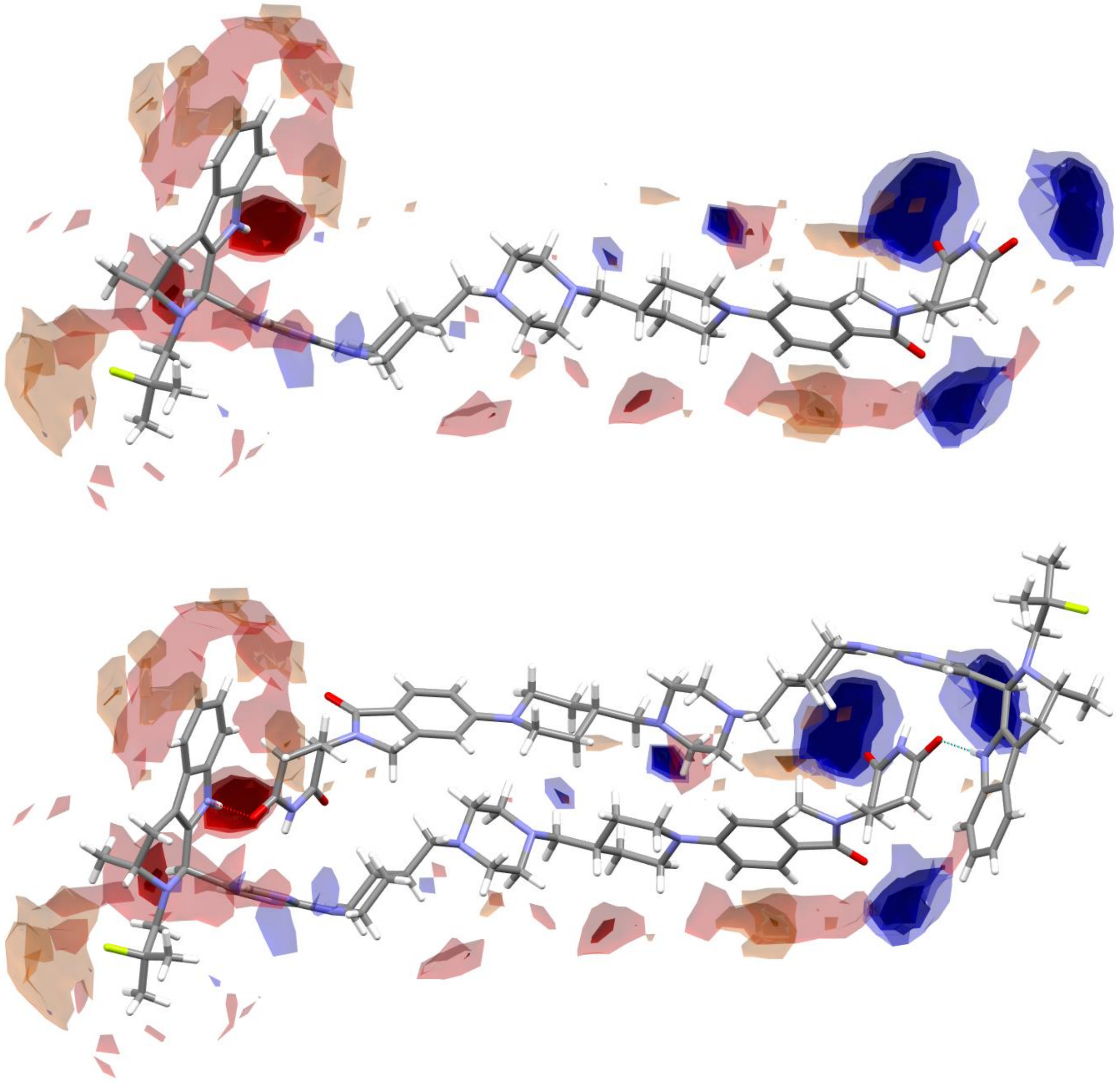

Figure **S6**. Full interaction maps of AZ1 Form 3 calculated using Mercury.[50] Red surfaces indicate a predicted probability of hydrogen-bond acceptors; blue surfaces indicate a predicted probability of hydrogen-bond donors; brown surfaces indicate a hydrophobic preference.

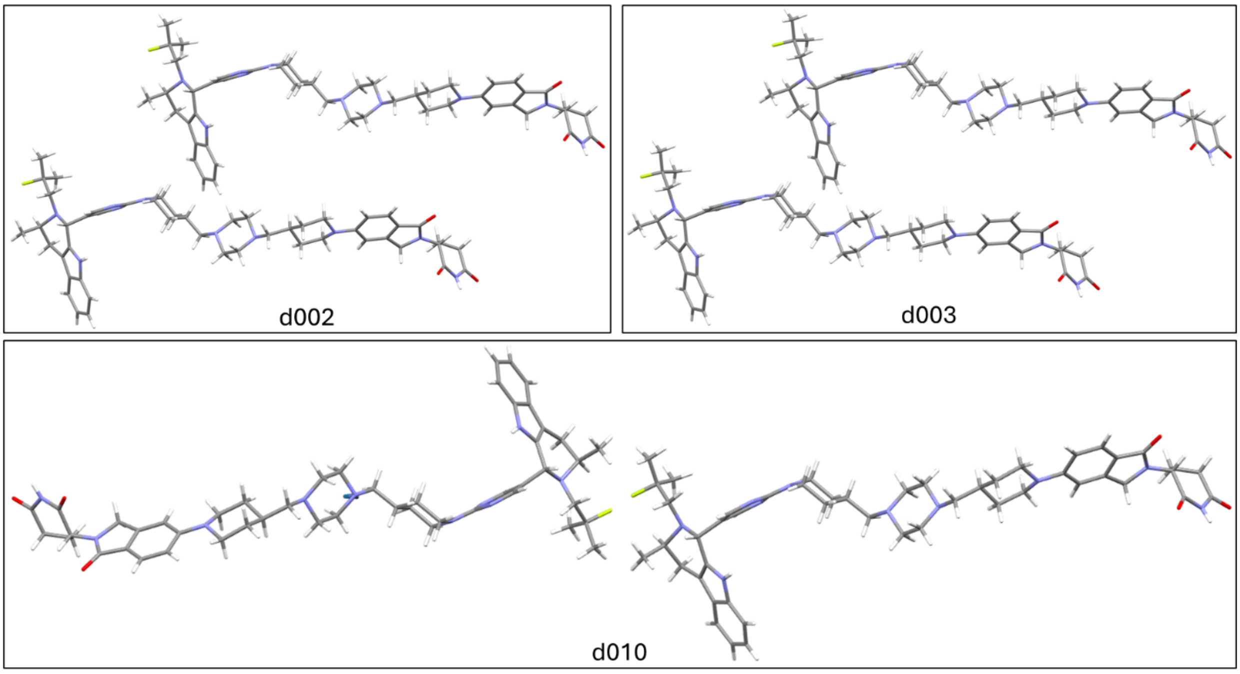

Figure **S7**. CrysIn interaction pairs for Form 3 not included in Figure 11.

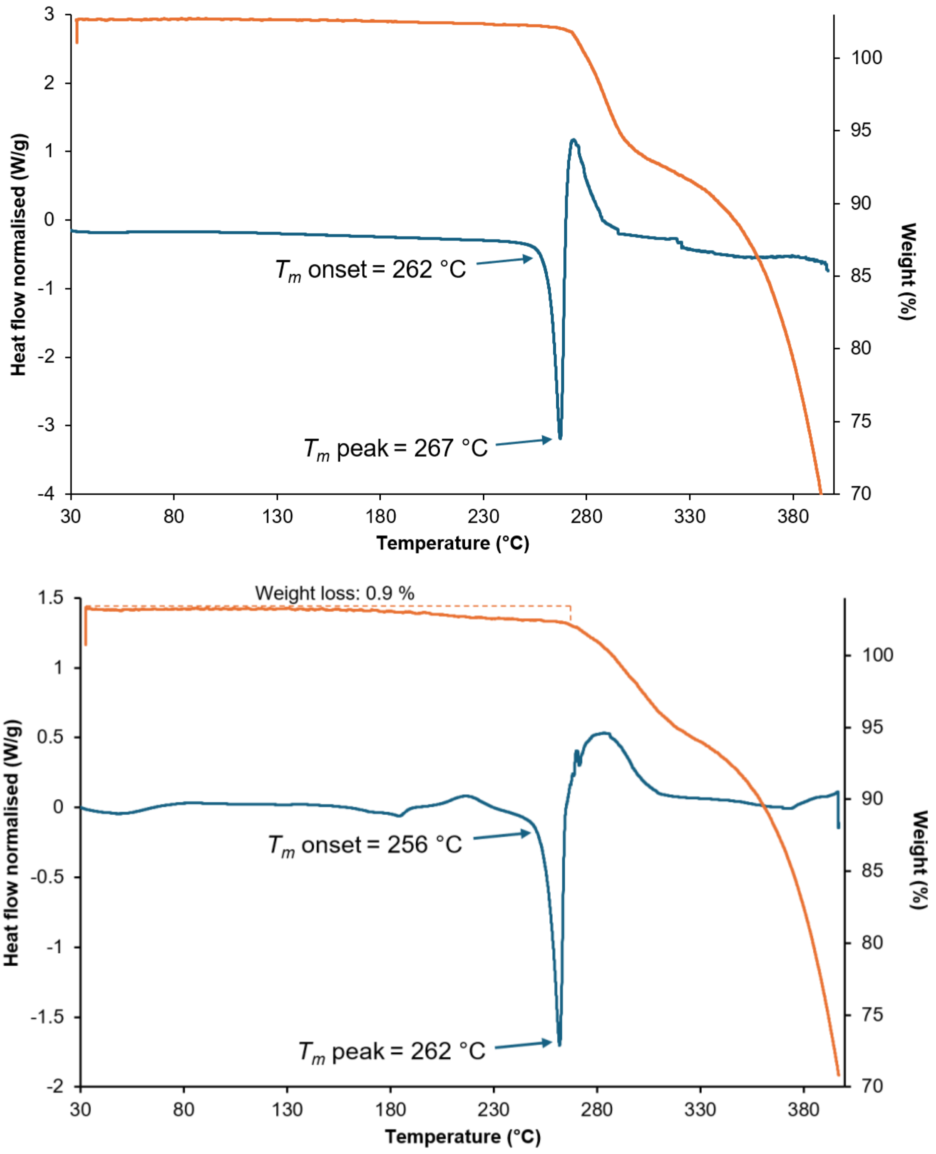

Figure **S8**. DSC and TGA of Form 1 $AZ1_{RRS}$ (top) and $AZ1_{mix}$ (bottom). Top) Melt decomposition onset at 262, peak at 267 °C. Bottom) 0.9% mass loss from ~190 up to 260 °C. Melt decomposition onset at 256, peak at 262 °C. While neither sample appears to contain any amorphous content by XRPD analysis, the DSC thermogram of Form 1 obtained with $AZ1_{mix}$ contains a broad exotherm-like feature between 150 and 200 °C that corresponds to a loss in mass of approximately 0.8 %, which could be a broad glass transition temperature (T_g) for some amorphous content dispersed through the sample. The $AZ1_{RRS}$ sample is completely dry by comparison. Since $AZ1_{mix}$ contains a 1:1 mixture of RRR- and RRS- isomers, the evidence of only a small quantity of amorphous content suggests it is highly unlikely that only one isomer crystallises as Form 1 while the remaining 50 % of the mass is the other isomer as completely amorphous solid. It is more likely that, like Form 3, both isomers are capable of packing into the same crystal structure but potentially to different extents.

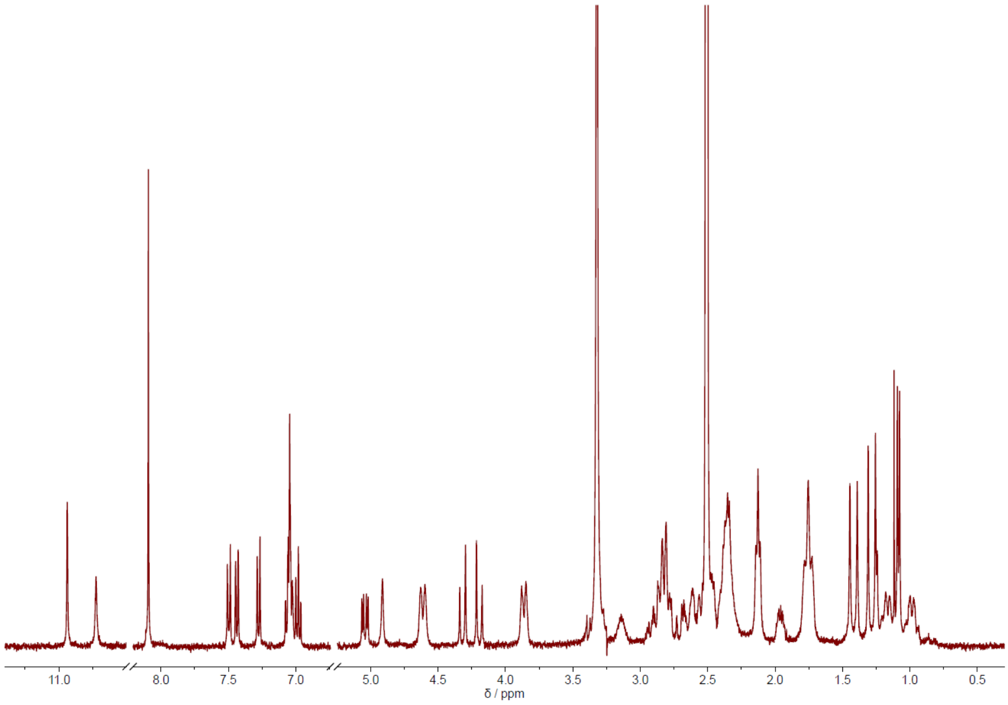

Figure **S9**. Solution-state ^{1}H NMR spectrum of Form 1 showing only residual DMSO and H2O solvent peaks.

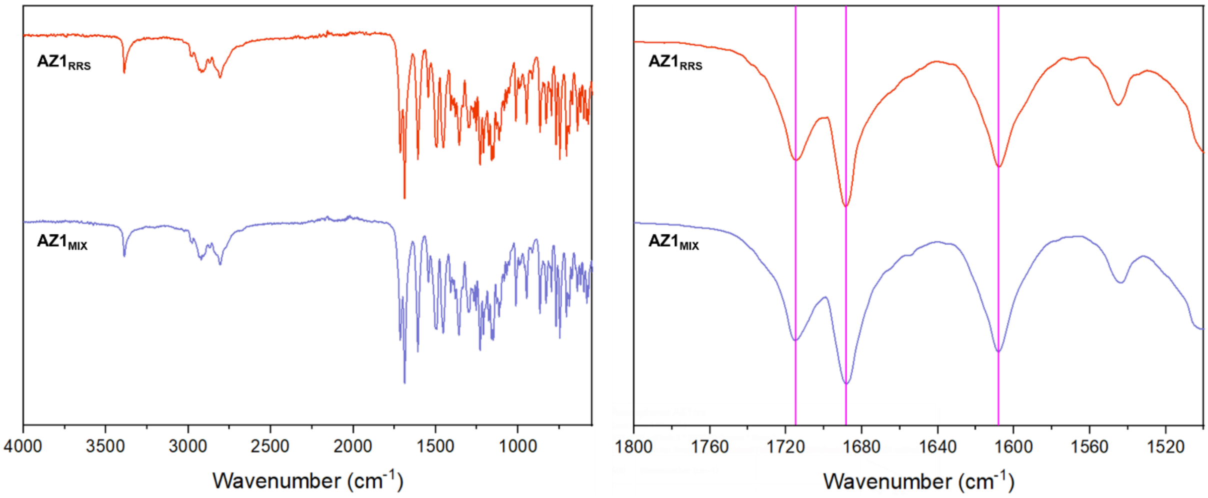

Figure **S10**. FTIR spectra of Form 1 prepared from $AZ1_{MIX}$ and $AZ1_{RRS}$, showing that both solids are indistinguishable.

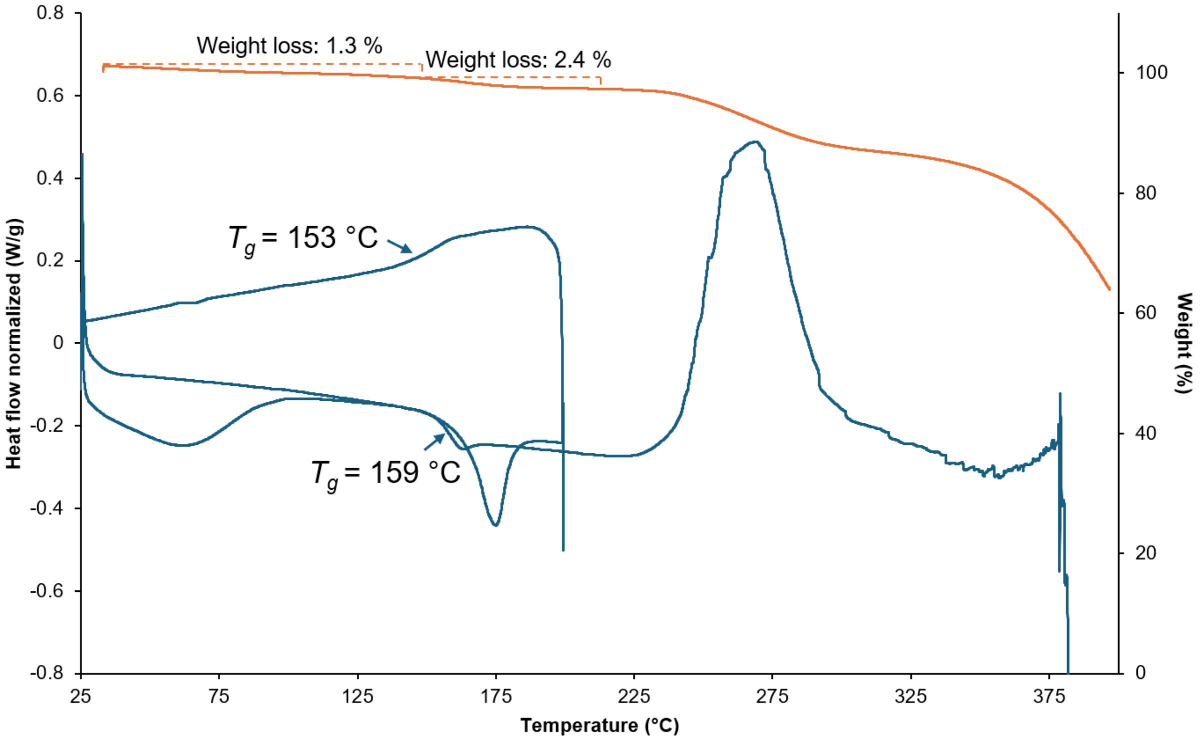

Figure **S11**. Heat-cool-heat DSC and TGA of Form 2 (DCM). Gradual loss of 1.3% mass up to 118 °C attributed to labile DCM, followed by 2.4% mass loss between ~120 and 200 °C attributed to desolvation of crystalline DCM. Desolvation leads to collapse of the crystal structure, with T_g values observed at 153 °C on cooling and 159 °C on reheating, followed by decomposition at 230 °C.

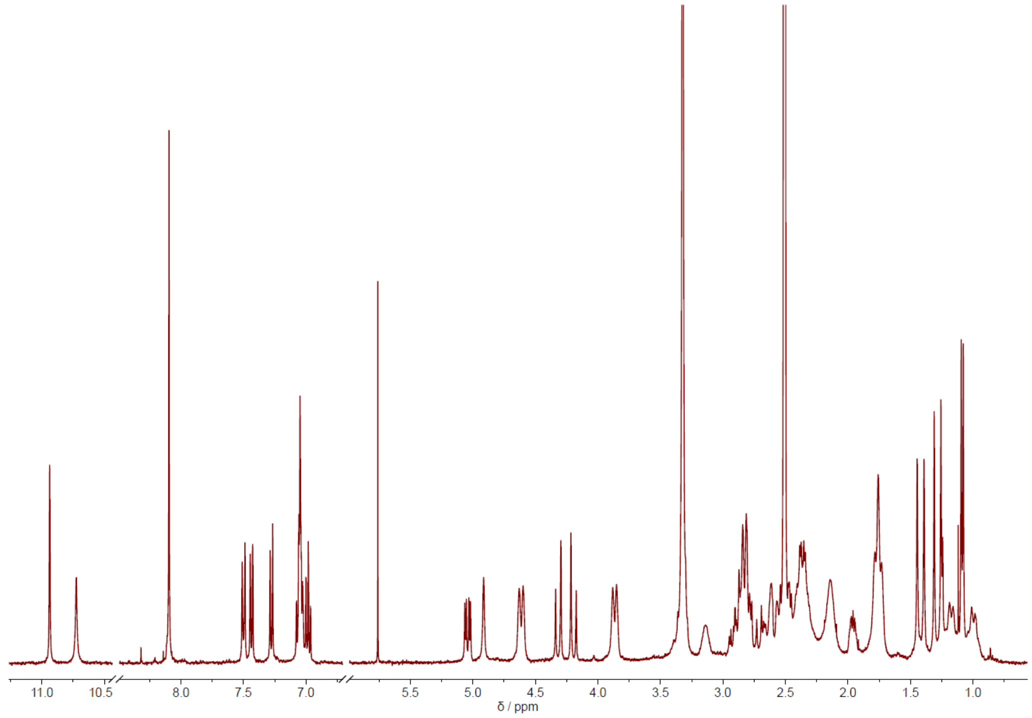

Figure **S12**. Solution-state ^{1}H NMR spectrum of Form 2 showing residual DMSO and H2O solvent peaks as well as a characteristic dichloromethane signal (singlet at 5.76 ppm).

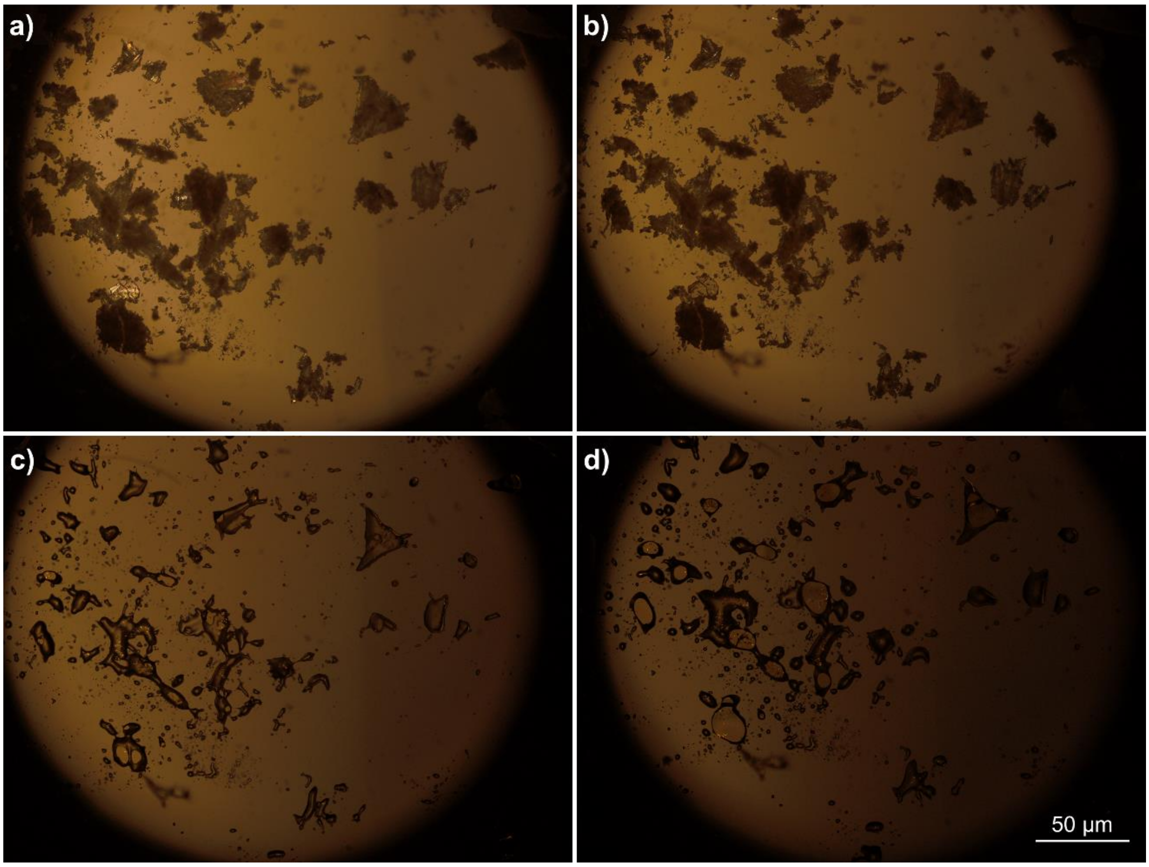

Figure **S13**. Hot-stage polarized optical microscopy (HS-POM) images of Form 2 ($AZ1_{mix}$) at a) 30 °C, b) 170 °C, c) 170 °C after holding for 10 minutes and d) 30 °C upon cooling. The sample was heated at 10 °C/min from 30 to 170 °C, held for 10 minutes and cooled back to 30 °C at 10 °C/min. The needle-like crystals appear to have been broken and clumped together during the preparation of the slide, with some shiny particles still evident at the start of the experiment. These appear to melt and/or desolvate around 170 °C and remain amorphous upon cooling.

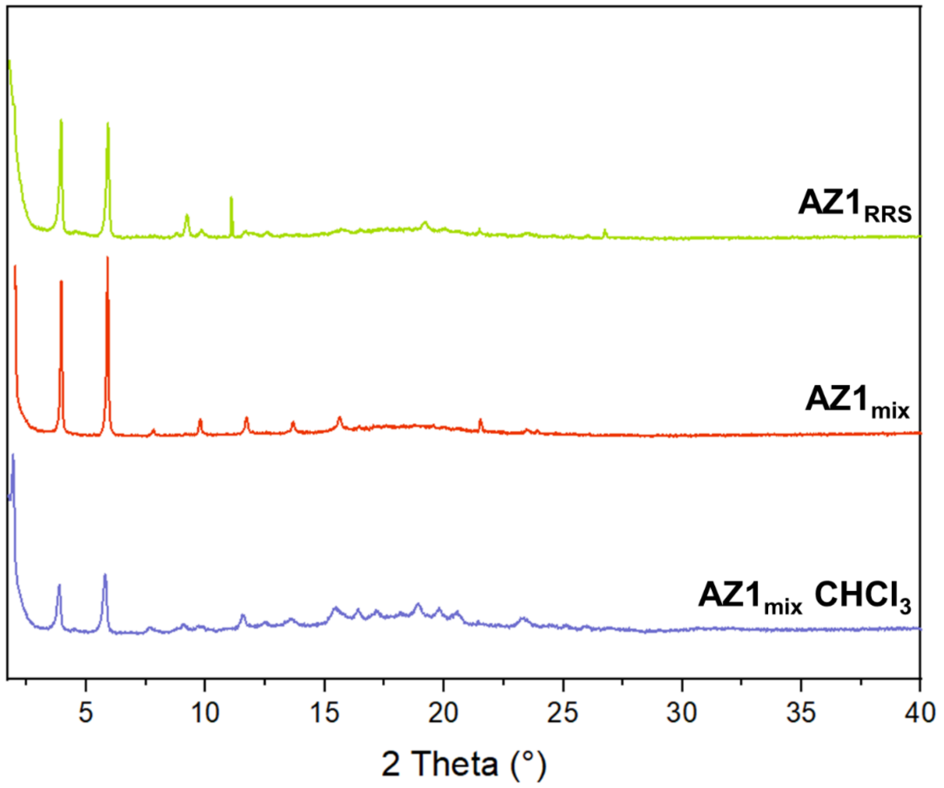

Figure **S14**. XRPD patterns for Form 2 produced using $AZ1_{RRS}$ (top) and $AZ1_{mix}$ (centre) and a sample crystallised from chloroform that appears to be isostructural.

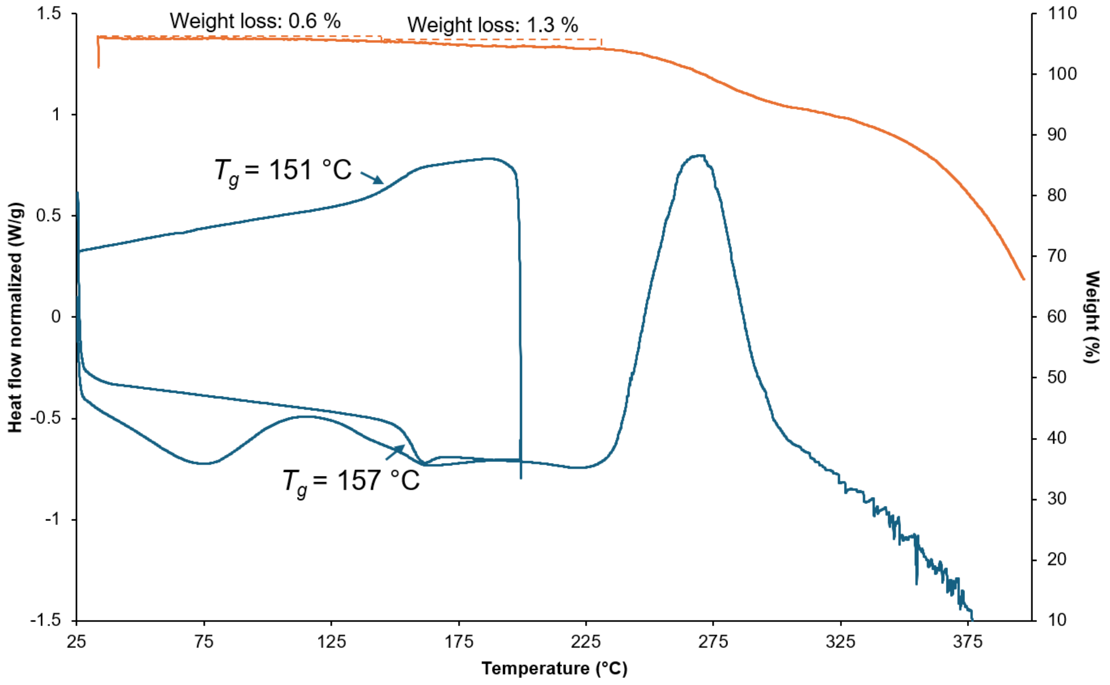

Figure **S15**. Heat-cool-heat DSC and TGA of Form 2 ($CHCl_3$). Gradual loss of 0.6% mass up to 130 °C attributed to labile chloroform, followed by 1.3% mass loss between ~155 and 225 °C attributed to desolvation of crystalline $CHCl_3$, for a combined total stoichiometry of 1 : 0.14 moles of AZ1 to $CHCl_3$. Desolvation leads to collapse of the crystal structure, with T_g values observed at 151 °C on cooling and 157 °C on reheating, followed by decomposition at 230 °C.

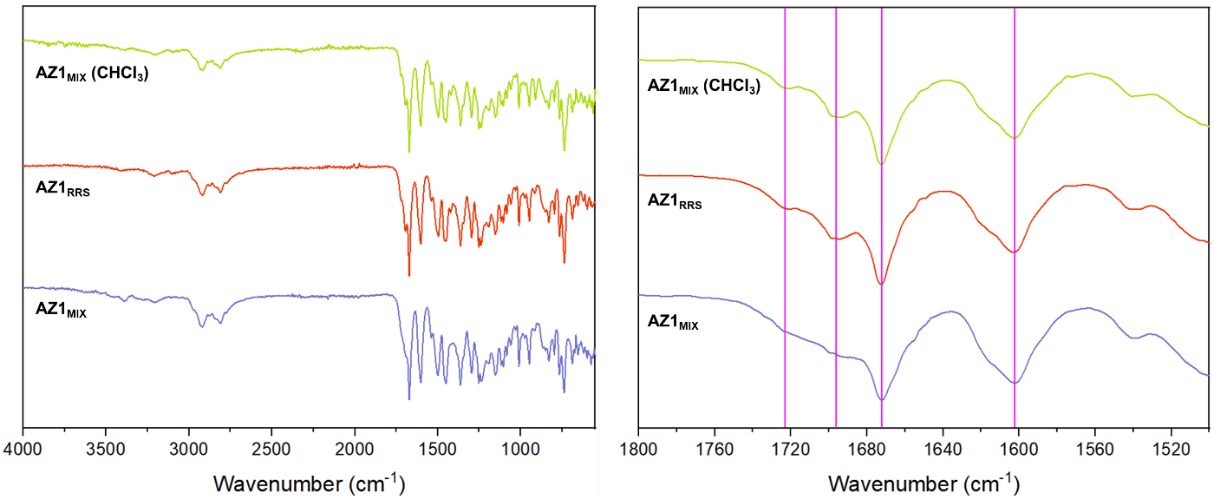

Figure **S16**. FTIR spectra for Form 2 (DCM) produced using $AZ1_{mix}$ (bottom), $AZ1_{RRS}$ (centre) and a potentially isostructural sample produced from chloroform (top). Form 2 (DCM) samples differ only in the sharpness of spectral features, with the $AZ1_{RRS}$ sample showing greater crystallinity as per Figure S11.

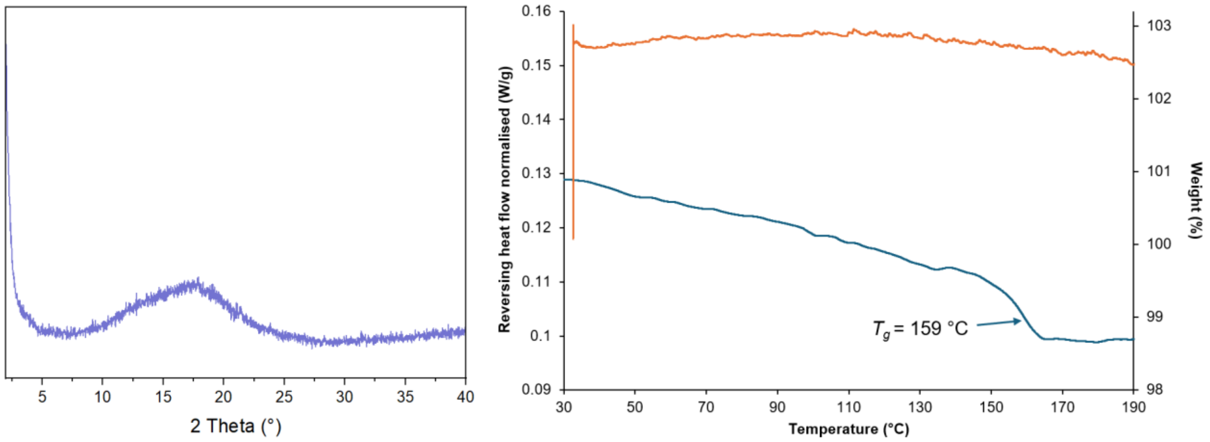

Figure **S17**. XRPD pattern and DSC/TGA of scaled-up amorphous form B, showing a T_g at 159 °C and no solvent loss up to 190 °C.

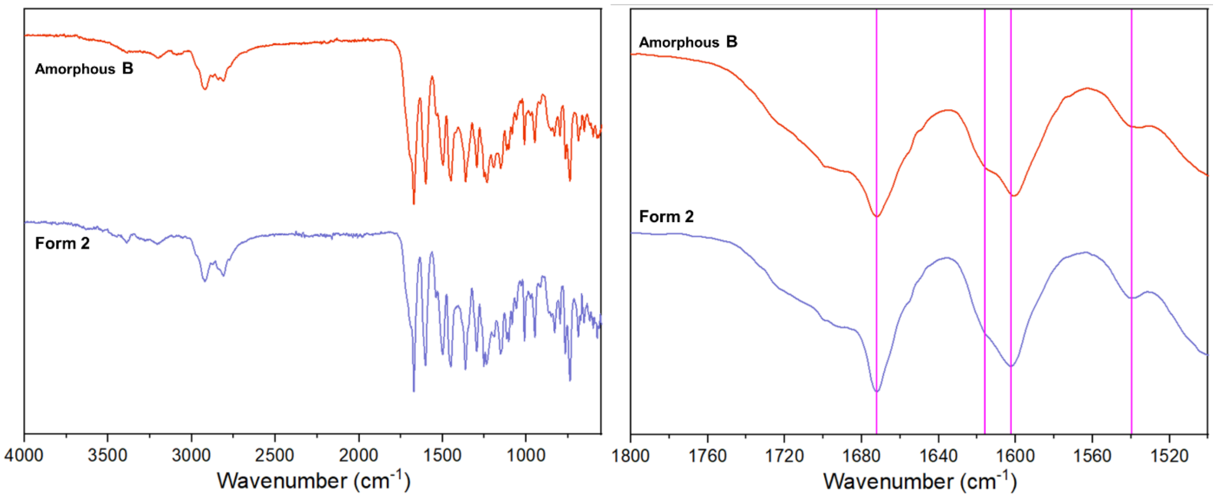

Figure **S18**. FTIR spectra comparing Form 2 and desolvated form 2 (amorphous form B). The spectra are indistinguishable.

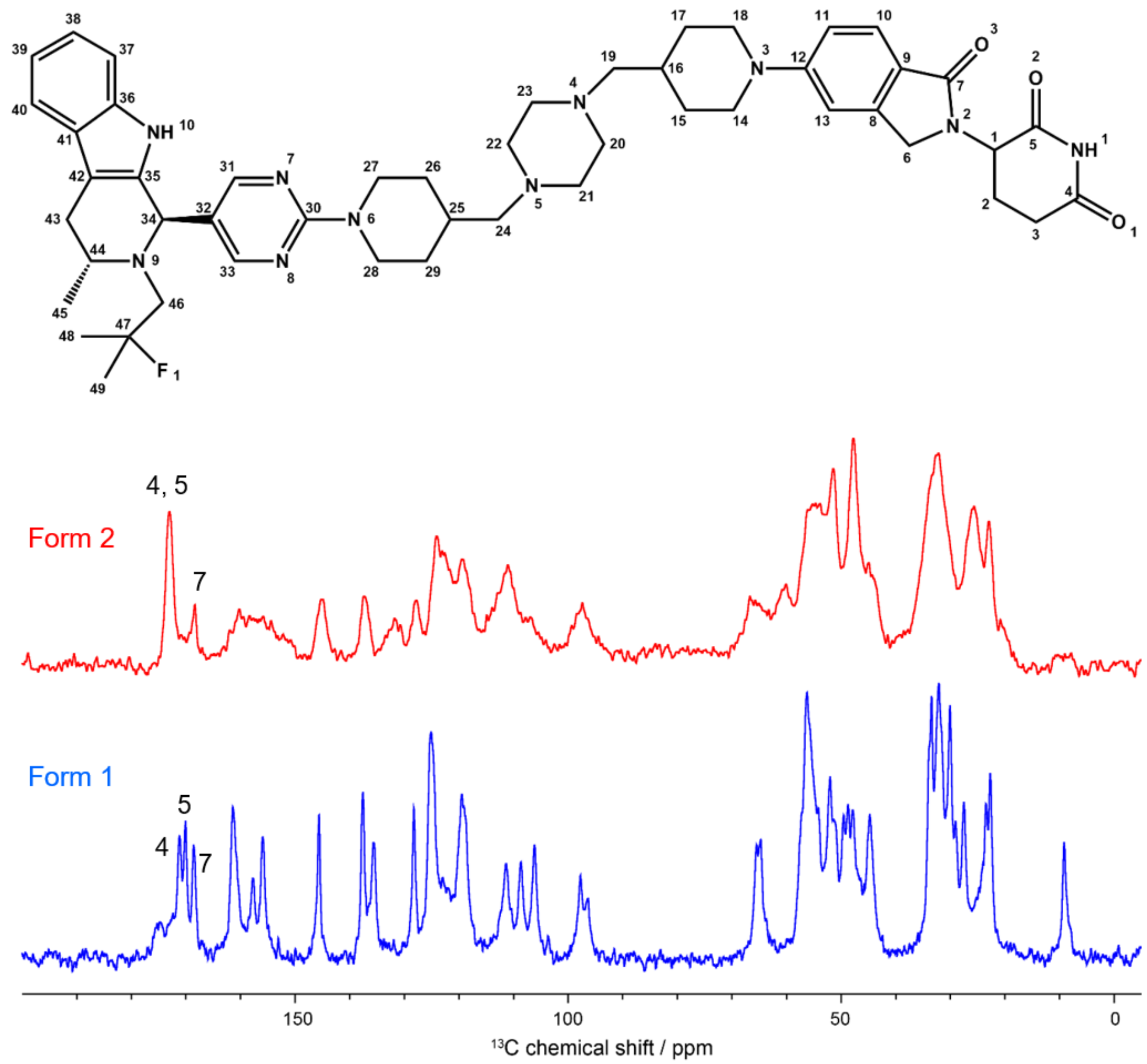

Figure **S19**. ^{13}C CPTOSS spectra of Form 1 ($AZ1_{RRS}$) and Form 2 ($AZ1_{mix}$) acquired with 512 and 3600 scans, and recycle delays of 4 s and 1 s, respectively, at a MAS rate of 10 kHz. Excitation and decoupling were achieved using a 3 μs ^{1}H 90° pulse corresponding to a nutation rate of 83.3 kHz. 25 and 40 Hz of Lorentzian line broadening was applied to Form 1 and 2 respectively. An atomic numbering scheme is shown above, indicating that carbon atoms 4, 5 and 7 correspond to carbonyl carbon atoms. These have been assigned with the aid of CASTEP DFT-predicted ^{13}C chemical shifts (see Figure S19) of the Form 1 crystal structure obtained via electron diffraction.

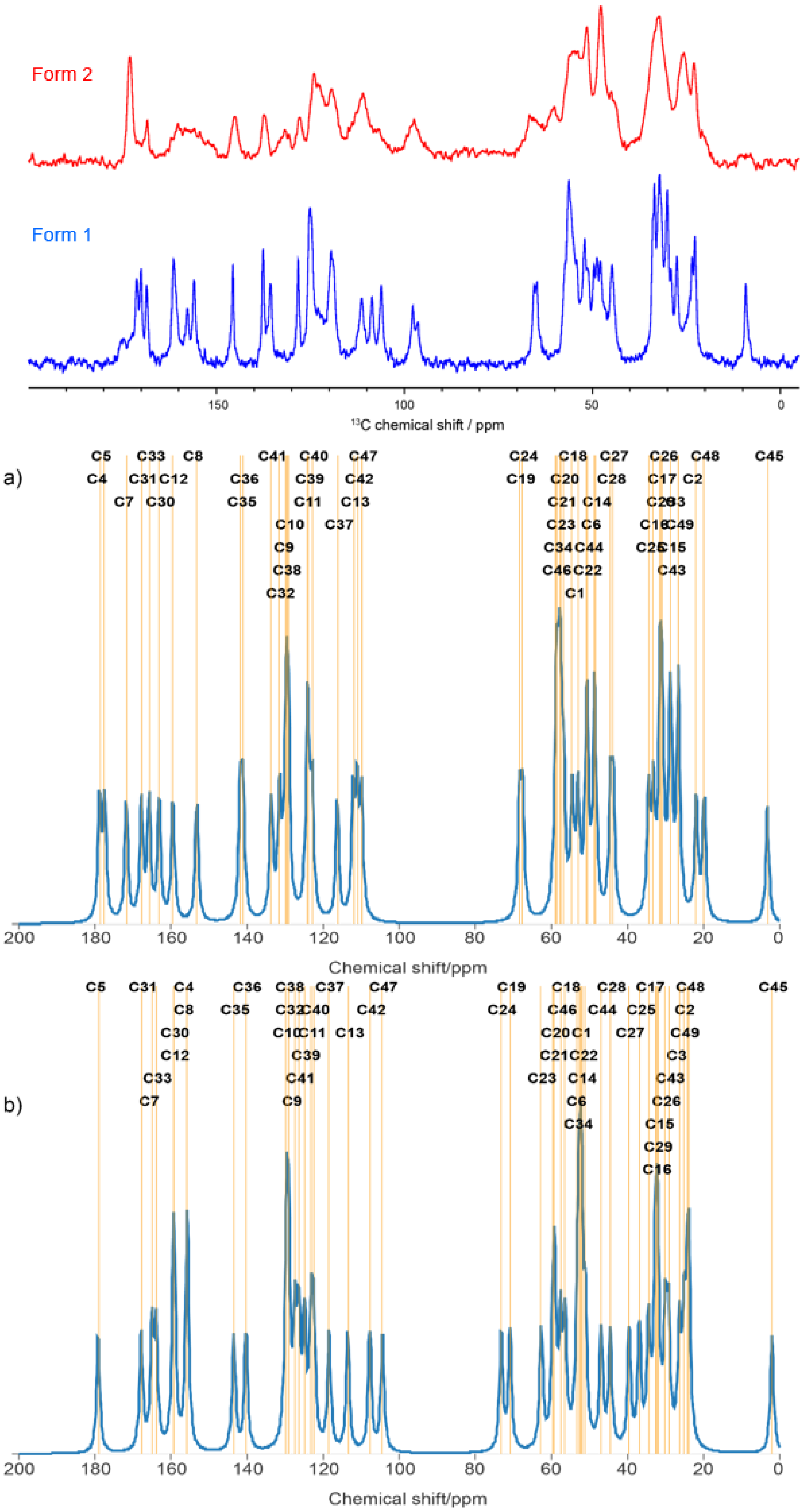
Form 2
Form 1
150
100
50
0
13C chemical shift / ppm
a)
C5 C33 C8 C41 C40 C47 C24 C18 C27 C26 C48 C45
C4 C31 C12 C36 C39 C42 C19 C20 C28 C17 C2
C7 C30 C35 C11 C13 C21 C14
C10 C37 C23 C6 C49
C9 C34 C44 C15
C38 C46 C22 C43
C32 C1
200 180 160 140 120 100 80 60 40 20 0
Chemical shift/ppm
b)
C5 C31 C4 C36 C38 C37 C47 C19 C18 C28 C17 C48 C45
C8 C35 C32 C40 C42 C24 C46 C44 C25 C2
C30 C10 C11 C13 C20 C1 C27 C49
C12 C39 C21 C22 C3
C33 C41 C23 C14 C43
C7 C9 C6 C26
C34 C15
C29
C16
200 180 160 140 120 100 80 60 40 20 0
Chemical shift/ppm

Figure **S20**. NMR prediction via CASTEP calculation of Form 1, in which all atomic positions have been geometry optimised a) and in which the heavy atoms have been frozen b). The predicted spectra have been compared versus the experimental spectra from Figure S18. There is a noticeable difference between the resultant predicted NMR spectra in the alkyl region (15–80 ppm), with the fully geometry optimised structure (a) bearing a closer resemblance to the experimental NMR spectrum. This would be consistent with a degree of disorder / thermal motion in the linker region of structure. The 3D ED structure of Form 1 was obtained at 175 K.

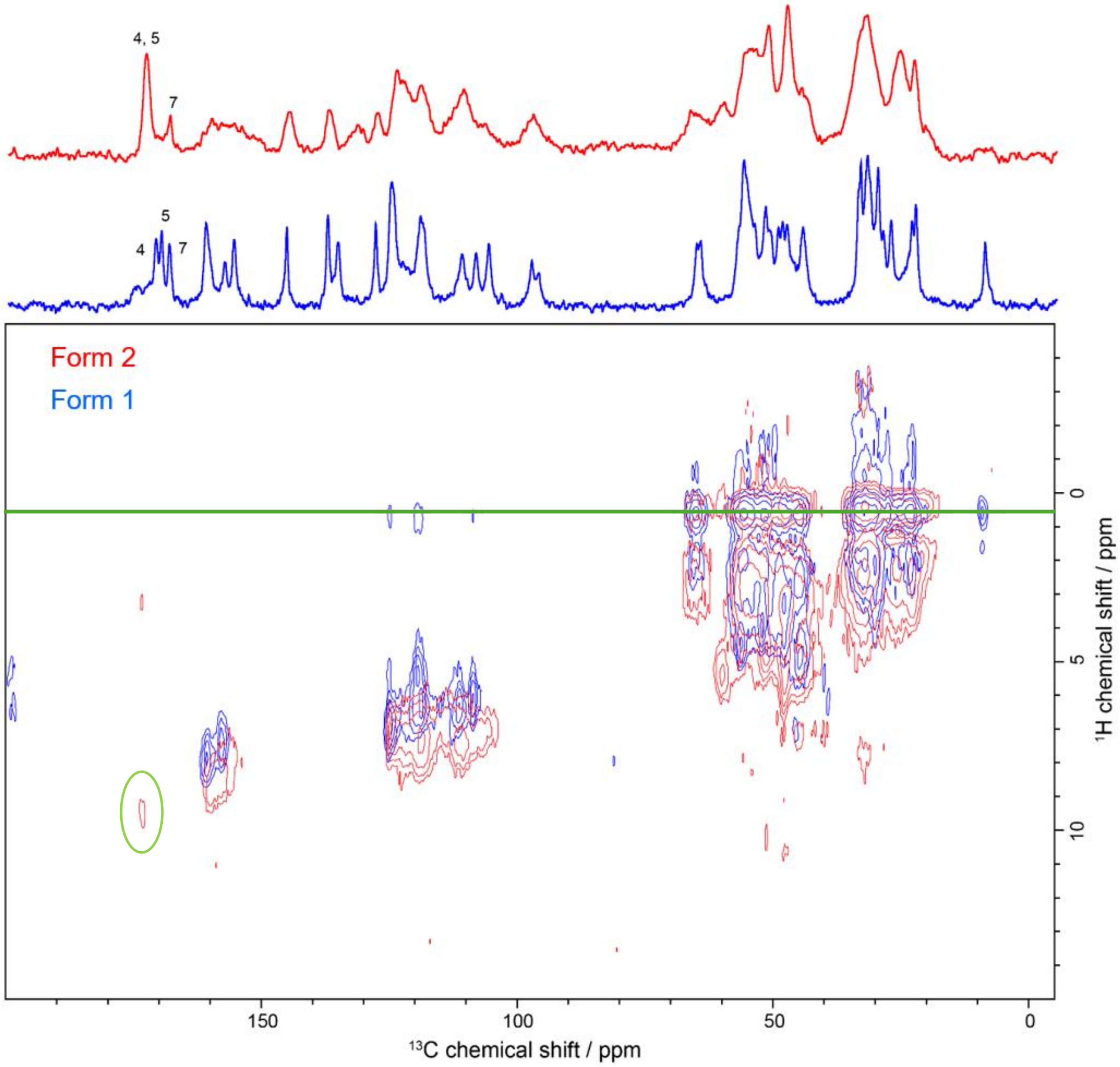

Figure **S21**. ^{1}H-^{13}C FSLG HETCOR spectra of Form 1 ($AZ1_{RRS}$) and Form 2 ($AZ1_{mix}$) acquired with a 100 µs contact pulse, 512 and 900 scans per slice, and recycle delays of 4 s and 1 s, respectively, at a MAS rate of 10 kHz. 80 slices in the indirect dimension were acquired. Excitation and decoupling were achieved using a 3 µs ^{1}H 90° pulse corresponding to a nutation rate of 83.3 kHz. 40 Hz of Lorentzian line broadening was applied to the F2 dimension and a 0.3 Hz QSINE sine bell shift of 3 was applied to the F1. The horizontal external projections are the CPTOSS spectra from Figure S18. The cross peaks denoted by the green horizontal line are an artifact resulting from the LG decoupling. The carbonyl peaks produce only a single weak contact in Form 2 at short contact times (highlighted by the green oval), corresponding to the interaction between C4 and C5 and what is most likely the N1H group, due its high chemical shift at 9.5 ppm.

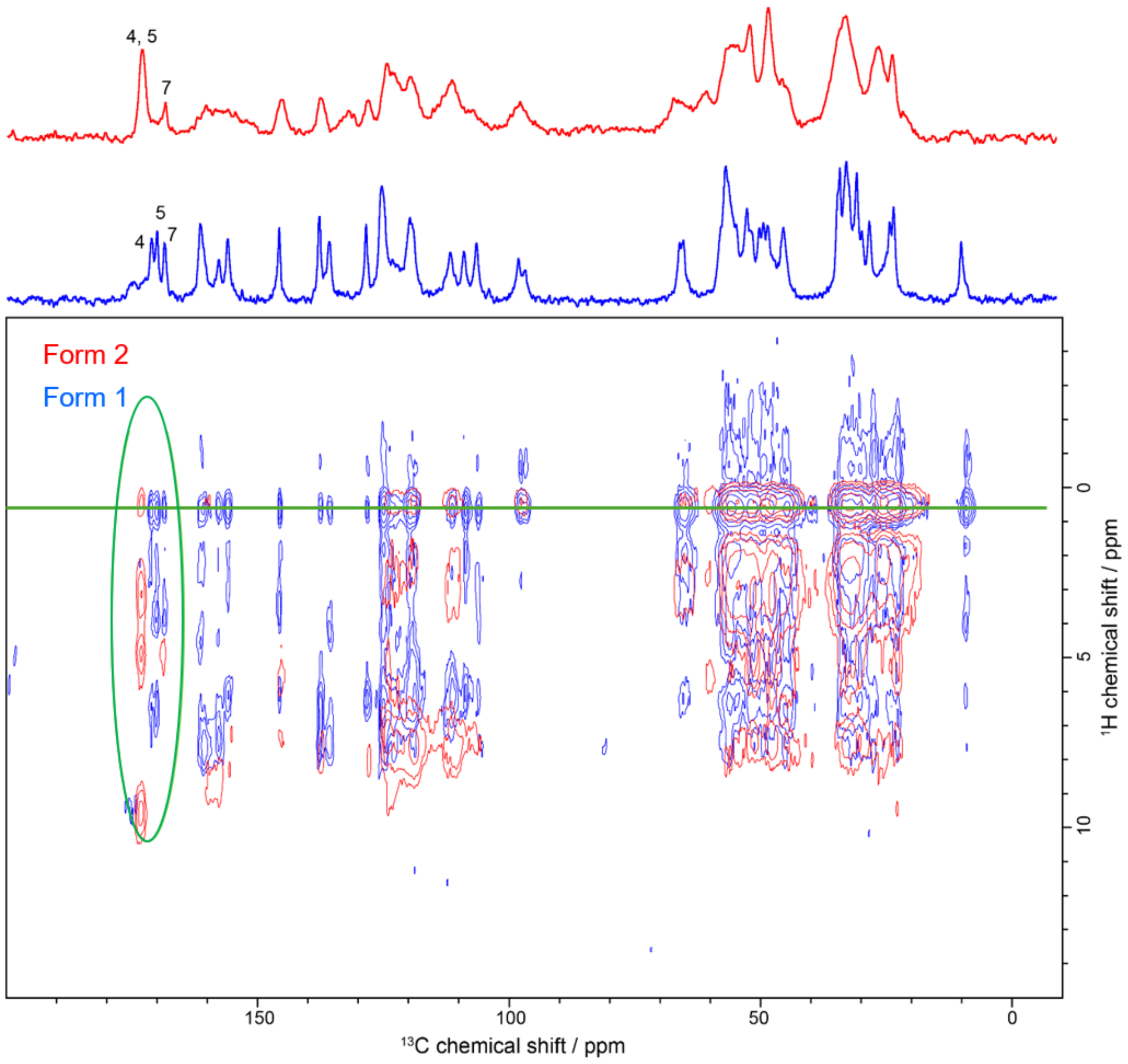

Figure **S22**. ^{1}H-^{13}C FSLG HETCOR spectra of Form 1 ($AZ1_{RRS}$) and Form 2 ($AZ1_{mix}$) acquired with a 1000 μs contact pulse, 512 and 900 scans per slice, and recycle delays of 4 s and 1 s, respectively, at a MAS rate of 10 kHz. 80 slices in the indirect dimension were acquired. Excitation and decoupling were achieved using a 3 μs ^{1}H 90° pulse corresponding to a nutation rate of 83.3 kHz. 40 Hz of Lorentzian line broadening was applied to the F2 dimension and a 0.3 Hz QSINE sine bell shift of 3 was applied to the F1. The horizontal external projections are the CPTOSS spectra from Figure S18. The cross peaks denoted by the green horizontal line are an artifact resulting from the LG decoupling. At longer contact times, the interaction between C4/C5 and N1H is much stronger, and longer distance dipolar interactions are also observed for both forms (highlighted by the green oval), which can be attributed to the interaction between the carbonyl carbon atoms and neighbouring alkyl hydrogen atoms, as the cross peaks appear at lower chemical shifts in the F1 dimension. These cross peaks likely correspond to the interaction between the carbonyl carbon atoms, C4 and C5, and protons on neighbouring carbon atoms on that same ring (such and C2 and C3), as well as the N1H group. There is also a cross peak corresponding to an interaction between the carbonyl C7 group and neighbouring protons. There does not appear to be any evidence of a long-distance interaction between the C4 and C5 carbonyl atoms and the N10H group on the other side of the molecule.

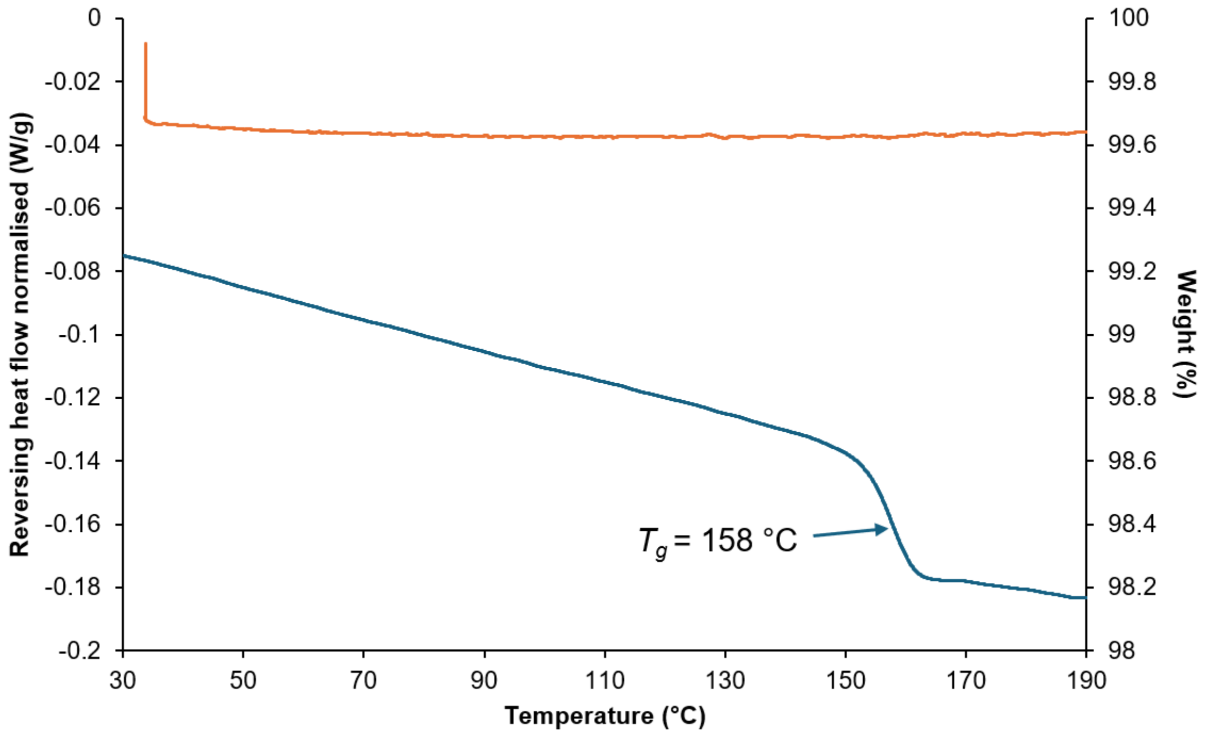

Figure **S23**. DSC/TGA of amorphous form A (AZ1$_{mix}$) showing a T_g at 158 °C and no solvent loss up to 190 °C.

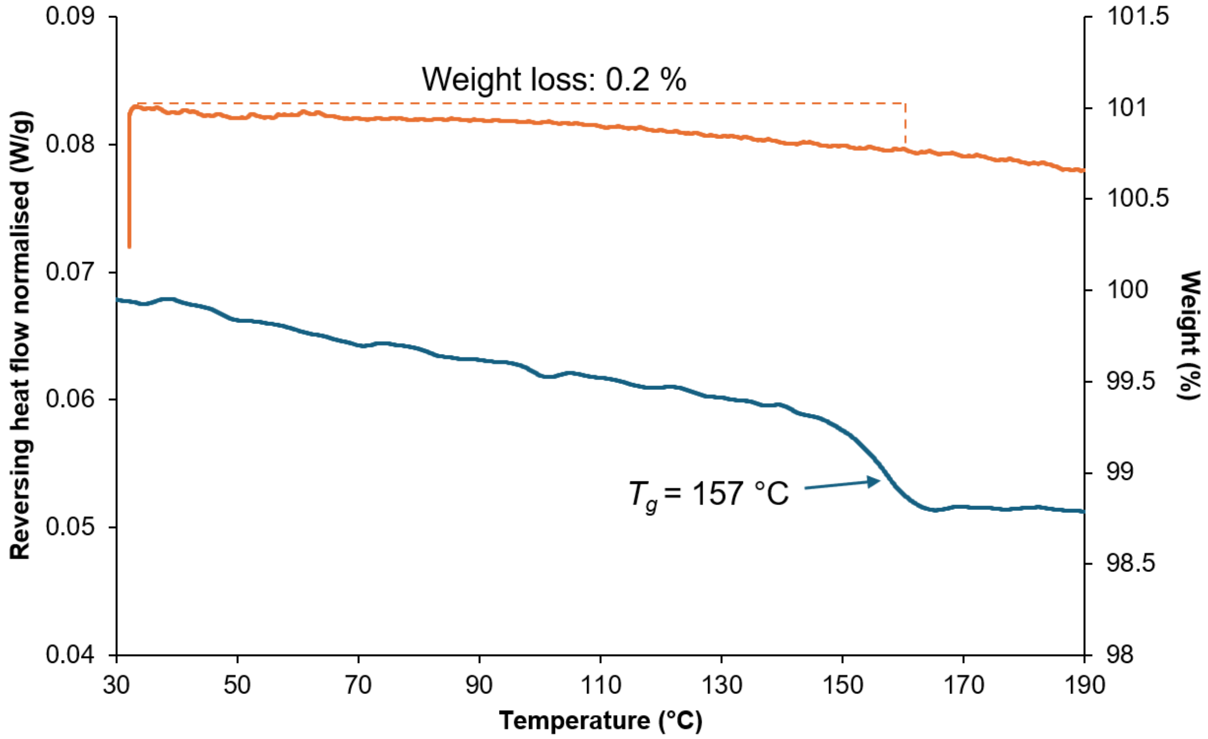

Figure **S24**. DSC/TGA of amorphous form A (AZ1$_{RRS}$) showing a T_g at 157 °C and 0.2 % weight loss up to 190 °C.

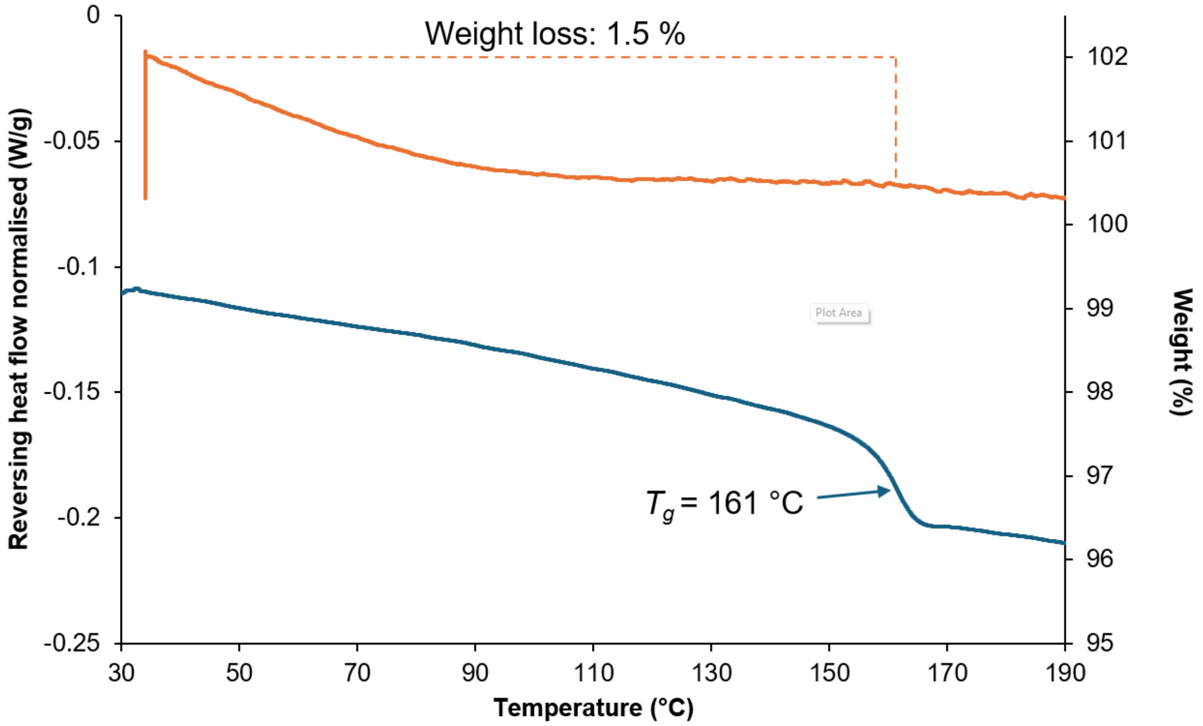

Figure **S25**. DSC/TGA of amorphous form A ($AZ1_{RRR}$) showing a T_g at 161 °C and 1.5 % weight loss up to 190 °C.
